# Growth Factor and Th2 Cytokine Signaling Pathways Converge at STAT6 to Promote Arginase Expression in Progressive Experimental Visceral Leishmaniasis

**DOI:** 10.1371/journal.ppat.1004165

**Published:** 2014-06-26

**Authors:** E. Yaneth Osorio, Bruno L. Travi, Alda M. da Cruz, Omar A. Saldarriaga, Audrie A. Medina, Peter C. Melby

**Affiliations:** 1 Department of Internal Medicine, University of Texas Medical Branch, Galveston, Texas, United States of America; 2 Laboratório Interdisciplinar de Pesquisas Médicas (LIPMED), Instituto Oswaldo Cruz-FIOCRUZ, Rio de Janeiro, Brazil; 3 Department of Microbiology and Immunology, University of Texas Medical Branch, Galveston, Texas, United States of America; 4 Center for Tropical Diseases, and Institute for Human Infection and Immunity, University of Texas Medical Branch, Galveston, Texas, United States of America; 5 Department of Pathology, and Sealy Center for Vaccine Development, University of Texas Medical Branch, Galveston, Texas, United States of America; University of Michigan, United States of America

## Abstract

Host arginase 1 (arg1) expression is a significant contributor to the pathogenesis of progressive visceral leishmaniasis (VL), a neglected tropical disease caused by the intracellular protozoan *Leishmania donovani*. Previously we found that parasite-induced arg1 expression in macrophages was dependent on STAT6 activation. Arg1 expression was amplified by, but did not require, IL-4, and required *de novo* synthesis of unknown protein(s). To further explore the mechanisms involved in arg1 regulation in VL, we screened a panel of kinase inhibitors and found that inhibitors of growth factor signaling reduced arg1 expression in splenic macrophages from hamsters with VL. Analysis of growth factors and their signaling pathways revealed that the Fibroblast Growth Factor Receptor 1 (FGFR-1) and Insulin-like Growth Factor 1 Receptor (IGF-1R) and a number of downstream signaling proteins were activated in splenic macrophages isolated from hamsters infected with *L. donovani*. Recombinant FGF-2 and IGF-1 increased the expression of arg1 in *L. donovani* infected hamster macrophages, and this induction was augmented by IL-4. Inhibition of FGFR-1 and IGF-1R decreased arg1 expression and restricted *L. donovani* replication in both *in vitro* and *ex vivo* models of infection. Inhibition of the downstream signaling molecules JAK and AKT also reduced the expression of arg1 in infected macrophages. STAT6 was activated in infected macrophages exposed to either FGF-2 or IGF-1, and STAT6 was critical to the FGFR-1- and IGF-1R-mediated expression of arg1. The converse was also true as inhibition of FGFR-1 and IGF-1R reduced the activation of STAT6 in infected macrophages. Collectively, these data indicate that the FGFR/IGF-1R and IL-4 signaling pathways converge at STAT6 to promote pathologic arg1 expression and intracellular parasite survival in VL. Targeted interruption of these pathological processes offers an approach to restrain this relentlessly progressive disease.

## Author Summary

Visceral leishmaniasis (VL), caused by the intracellular protozoan *Leishmania donovani*, is a progressive infection that is particularly common in impoverished populations of the world. People die from this disease unless it is treated. We used an experimental infection model that mimics the clinical and pathological features of human VL to study how the parasite causes this severe disease. We found that host macrophages infected with *Leishmania donovani* are activated in a way that leads to the expression of arginase, an enzyme that counteracts the cell's mechanisms that control the infection. This disease-promoting activation pathway was driven by the convergence of growth factor and cytokine signaling pathways and activation of the transcription factor STAT6. Chemical inhibition of signaling through the fibroblast growth factor receptor-1 (FGFR-1) or insulin-like growth factor-1 receptor (IGF-IR), or genetic knockdown of STAT6 led to reduced expression of arginase and enhanced control of the infection by macrophages. This indicates that the growth factor signaling pathways together with the cytokine pathways promote this disease. Interventions designed to disrupt this signaling could help in the treatment of VL.

## Introduction

Visceral leishmaniasis (VL), caused by the intracellular protozoan *Leishmania donovani or L. infantum*, is one of the “Neglected Tropical Diseases” that impacts the poor of the world. Active VL is characterized by a relentlessly progressive infection with cachexia, massive splenomegaly, pancytopenia and ultimately death. VL ranks second to malaria in deaths caused by a protozoal pathogen; mortality is reported in up to 10–20% of patients, even with treatment [1]. The determinants of susceptibility and progressive disease are incompletely defined. However, it is clear that ineffective cellular immune function, dictated by the nature of cytokine response and polarization of macrophages [2], plays a critical role. Macrophages, the primary target of intracellular *Leishmania* infection, may take on distinct phenotypes in response to parasite signals and inflammatory stimuli within the infected microenvironment. Classically activated (M1) macrophages respond to IFN-γ and microbial products by generating antimicrobial molecules that effectively kill *Leishmania* and other intracellular pathogens [3], [4]. Central to the killing of intracellular parasites is the production of nitric oxide by the action of inducible nitric oxide synthase 2 (NOS2) on the substrate L-arginine. In contrast, alternatively activated or M2 macrophages, which are typically generated by exposure to type 2 cytokines (IL-4, IL-13), fail to produce antimicrobial effector molecules to kill intracellular pathogens and serve to dampen inflammation and promote wound healing [5], [6].

The activation status of macrophages in human VL has not been directly investigated. However, the progressive nature of the infection in the face of strong expression of IFN-γ [7]–[10], suggests that there is ineffective classical activation. The concomitant production of IL-4/IL-13 and IL-10 [7], [8], [11]–[14], which are known to impair macrophage leishmanicidal activity, may polarize macrophages toward a disease-promoting M2 phenotype. Neutralization of IL-10 in *ex vivo* splenocyte cultures from patients with VL promoted parasite clearance [15], but the importance of IL-4 and/or IL-13 in the pathogenesis of human VL is not clear. Additionally, *Leishmania*-driven subterfuge of a number of signaling pathways can render the macrophage less responsive to activating stimuli and more permissive to infection [16].

We have used the hamster model of VL, which closely mimics the clinicopathological features of human VL, to dissect the mechanisms by which *L. donovani* causes progressive disease. We demonstrated, similar to human VL, that progressive, lethal disease occurred in the face of what would be considered a protective type 1 cytokine response [17], [18]. Despite high expression of IFN-γ, it was ineffective in mediating classical activation of M1 macrophages and control of *Leishmania* infection. In fact we found that splenic macrophages from hamsters with VL were polarized to a M2-like phenotype with dominant expression of host arginase 1 (arg1) [2]. *L. donovani* triggered arg1 expression through a STAT6-dependent mechanism, but surprisingly it did not require type 2 cytokines [2]. Arginase contributes to intracellular *Leishmania* replication by competing with NOS2 for the substrate arginine (thereby reducing NO production), and by driving the generation of polyamines, which promote parasite growth [2], [19], [20]. M2-like macrophages and arginase have also been implicated in the pathogenesis of experimental cutaneous leishmaniasis [19]–[23] and infections with other intracellular pathogens [24]–[27]. Furthermore, there is accumulating evidence that arginase has a role in the pathogenesis of human disease. Although, polarization of isolated human macrophages by exposure to IL-4 *in vitro* did not lead to upregulation of arginase activity or arg1 expression [28], the presence of M2-like monocytes/macrophages and arginase expression has been found in cancer [29], [30], filariasis [25], tuberculosis [31], [32], and traumatic tissue injury [33]. Elevated arginase activity was also recently reported in the lesions of patients with chronic cutaneous leishmaniasis [34] and arginase expression in peripheral blood leukocytes was found to be a marker of active VL [35].

In this work we have investigated the mechanisms of the pathological upregulation of arg1 in the hamster model of progressive VL. We discovered that the expression of arg1 in *L. donovani* infected macrophages is driven by activation of fibroblast growth factor receptor (FGFR) and insulin-like growth factor-1 receptor (IGF-IR). Inhibition of these growth factor signaling pathways led to reduced arg1 expression and enhanced control of parasite replication. Furthermore, signaling molecules downstream of the growth factor receptors converged with IL-4 signaling to promote STAT6 activation and arg1 expression in VL. The intersection of these pathways leads to subversion of macrophage effector function and impaired host defense against VL.

## Results

### Receptor tyrosine kinases (RTK) are involved in parasite-induced arginase expression

We previously determined that *L. donovani* induced STAT6-dependent, host arg1 expression. Host arginase expression promoted parasite replication, so we sought to understand the mechanisms by which it was expressed in VL. Arg1 transcription required the *de novo* synthesis of protein [2] suggesting that transcription of arg1 involved signaling pathway(s) other than just direct phosphorylation of STAT6. We postulated that the newly synthesized protein could mediate its effect through RTK signaling pathways, which regulate inflammation and wound repair [36], [37]. Both of these processes are important functions of M2 macrophages. Therefore, we screened a library of 80 RTK inhibitors for inhibition of *L. donovani*-induced arginase transcription in an *ex vivo* model of infected splenocytes isolated from hamsters with VL [38]. Inhibitors of the Epidermal Growth Factor Receptor and Platelet-derived Growth Factor Receptor signaling pathways reduced arg1 transcription by >50% (Table 1). Because the RTK signaling pathways are overlapping and broad, and inhibitors of some growth factor receptors were not included in the inhibitor library, we used a RTK antibody array to further define the participation of specific RTKs in VL. We found that Fibroblast Growth Factor Receptor (FGFR) 1 and 2 and other molecules known to participate in growth factor signaling (Insulin receptor substrate 1 (IRS-1), v-akt murine thymoma viral oncogene homolog 1 and 2 (AKT 1/2), Mitogen-activated protein kinase (MAPK)-3, and Signal transducer and activator of transcription (STAT)-1, and STAT-3 were activated in splenic macrophages from hamsters infected with *L. donovani* (Table 2). Collectively, these data indicated that signaling through growth factor receptor pathways could contribute to the parasite-induced expression of host arg1.

**Table 1:** Inhibitors of Receptor Tyrosine Kinases reduce Arg1 transcription.

Target	Inhibitor	Concentration (µM)^1^	Arg1 Inhibition (%)^2^
EGFRK	Tyrphostin 25	6	64.6
EGFRK	Tyrphostin23	70	84.5
PDGFRK	Tyrphostin 9	2.4	78.5
EGFRK, PDGFRK	AG-494	46	62
MEK	U-0126	0.13	73.5
MEK	PD-98059	4	52.2
MAPK-p38, PKA, GSK-3-beta	Rottlerin	100	52.4
PI 3-K	Quercetin	7.6	83.5

1 Inhibitors (dissolved in DMSO) were used at twice the concentration reported to cause 50% inhibition of kinase activity.

No attempt was made to maximize concentration for complete kinase inhibition.

2 Arg1 mRNA expression was determined in ex vivo cultured spleen cells from hamsters infected with *L. donovani* exposed to the RTK inhibitor for 24 hrs.

The percent inhibition of arg1 transcription was calculated with reference to untreated control cells exposed to DMSO and determined by qPCR.

**Table 2:** Activation of Receptor Tyrosine Kinases and downstream signaling proteins in splenic macrophages from hamsters infected with *L. donovani*.^1^

Phosphoprotein (Entrez Gene Name)	Symbol	Fold Increase^2^
v-akt murine thymoma viral oncogene homolog 1	AKT1	20.7±12.1
v-akt murine thymoma viral oncogene homolog 2	AKT2	159.9±22.15
EPH receptor A1	EPHA1	7±3.95
EPH receptor A3	EPHA3	5.7±5.25
EPH receptor B4	EPHB4	4.9±0.3
Fibroblast growth factor receptor 1	FGFR1	2.1±0.35
Fibroblast growth factor receptor 3	FGFR3	2.7±0.3
Insulin receptor substrate 1	IRS1	40.2±16.3
v-kit Hardy-Zuckerman 4 feline sarcoma viral oncogene homolog	KIT	4.4±3.55
Mitogen-activated protein kinase 3	MAPK3	2.1±1.65
Neurotrophic tyrosine kinase, receptor, type 1	NTRK1	4.7±2.15
Neurotrophic tyrosine kinase, receptor, type 2	NTRK2	1.9±0.95
v-src sarcoma (Schmidt-Ruppin A-2) viral oncogene homolog (avian)	SRC	152±23.1
Signal transducer and activator of transcription 1	STAT1	5.7±2.45
Signal transducer and activator of transcription 3	STAT3	2.4±0.85
TEK tyrosine kinase, endothelial	TEK	7.3±3.65
zeta-chain (TCR) associated protein kinase 70 kDa	ZAP70	14±11.2

1 Determined using a PathScan Antibody Array (Chemiluniscent readout, Cell Signaling; 4 arrays per group) and analyzed with the IPA Software.

2 Fold-change (mean ± SEM) of the phosphoprotein expressed in splenic macrophages isolated from 28-day infected hamsters compared to splenic macrophages from uninfected hamsters.

RTKs and signaling proteins with a fold change >1.5 are shown.

### Growth factors induce arg1 in *L. donovani* infected hamster macrophages

**Figure 1:**
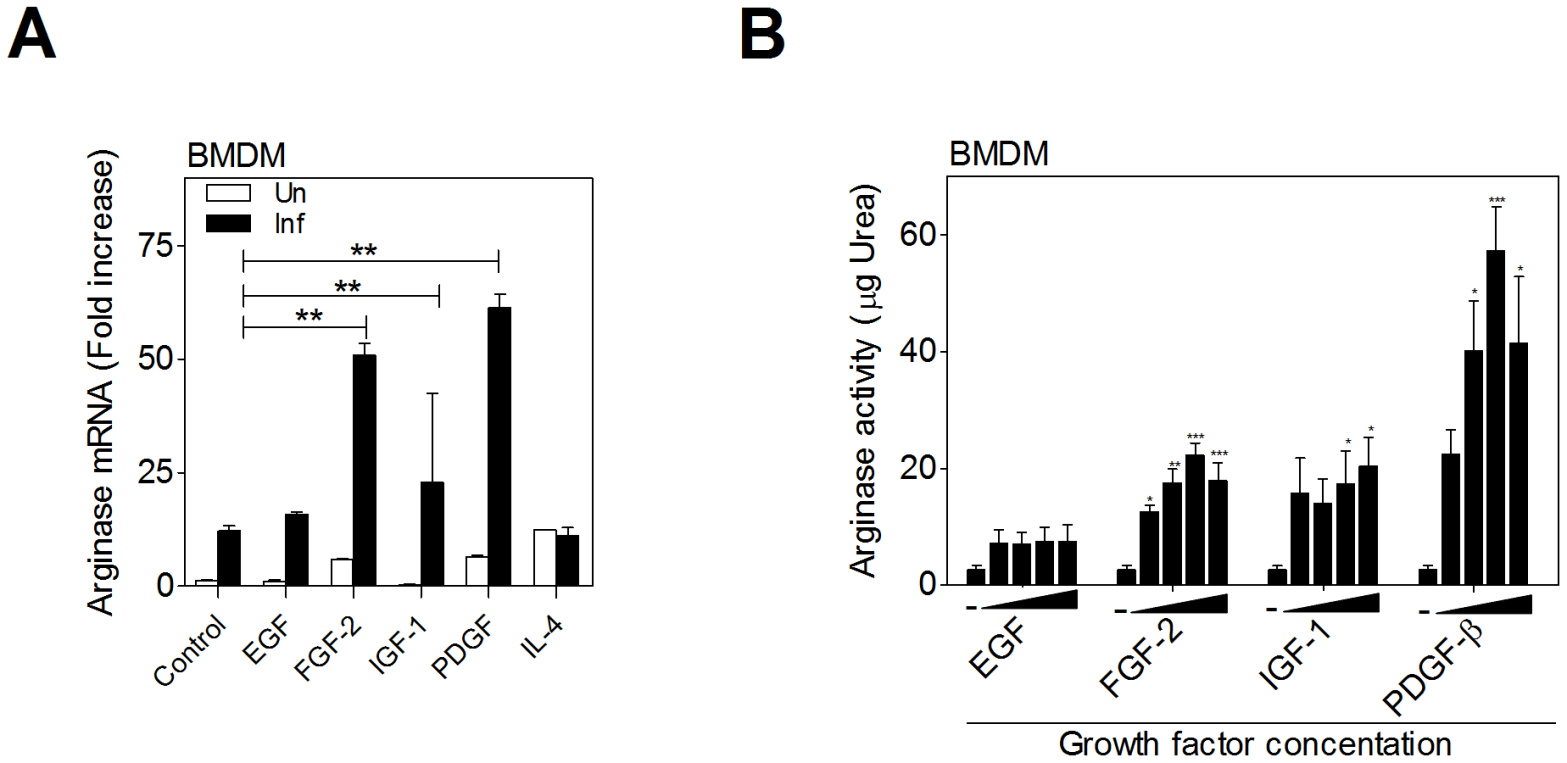
Growth factors upregulate arginase 1 in macrophages. **A**) Induction of arg1 mRNA expression in macrophages exposed to recombinant growth factors. Uninfected and *L. donovani* infected hamster BMDMs were stimulated with EGF (100 ng/mL), FGF-2 (20 ng/mL), IGF-1 (100 ng/mL), PDGF (100 ng/mL), or IL-4 (25 IU/mL) for 24 hrs and the expression of arg1 mRNA determined by qRT-PCR. Shown is the mean and standard error of the mean (SEM; error bars) of 4 replicates from a single experiment that is representative of 2 independent experiments. **B**) Dose-dependent induction of arginase activity (urea production) in hamster BMDMs infected with *L. donovani* and exposed to 2-fold increasing concentrations of growth factors for 48 h. The concentration of the growth factors was: EGF: 12.5–100 ng/mL; FGF-2: 6.25–50 ng/mL; IGF-1: 50–400 ng/mL; and PDGF: 25–100 ng/mL. Shown is the mean and SEM of 2 replicates per dose that is representative of 4 independent experiments. *p<0.05; **p<0.01; ***p<0.001.

A significant increase in arg1 mRNA expression was observed in *L. donovani* infected hamster bone marrow-derived macrophages (BMDM) exposed to the recombinant growth factors FGF-2, IGF-1, and PDGF (Fig. 1A). Growth factor-induced arg1 was particularly evident in infected compared to uninfected macrophages, and it was equivalent to, or greater than, IL-4-induced arg1. Arginase protein activity was also significantly increased in *L. donovani* infected BMDM exposed to FGF-2, IGF-1, and PDGF (Fig. 1B). EGF did not consistently induce a significant increase arg1 mRNA or protein. Together, these data suggested that *L. donovani* infection of macrophages led to enhanced arg1 transcriptional responsiveness to multiple growth factors.

### The FGF signaling pathway is activated in the spleens of hamsters infected with *Leishmania donovani*

**Figure 2:**
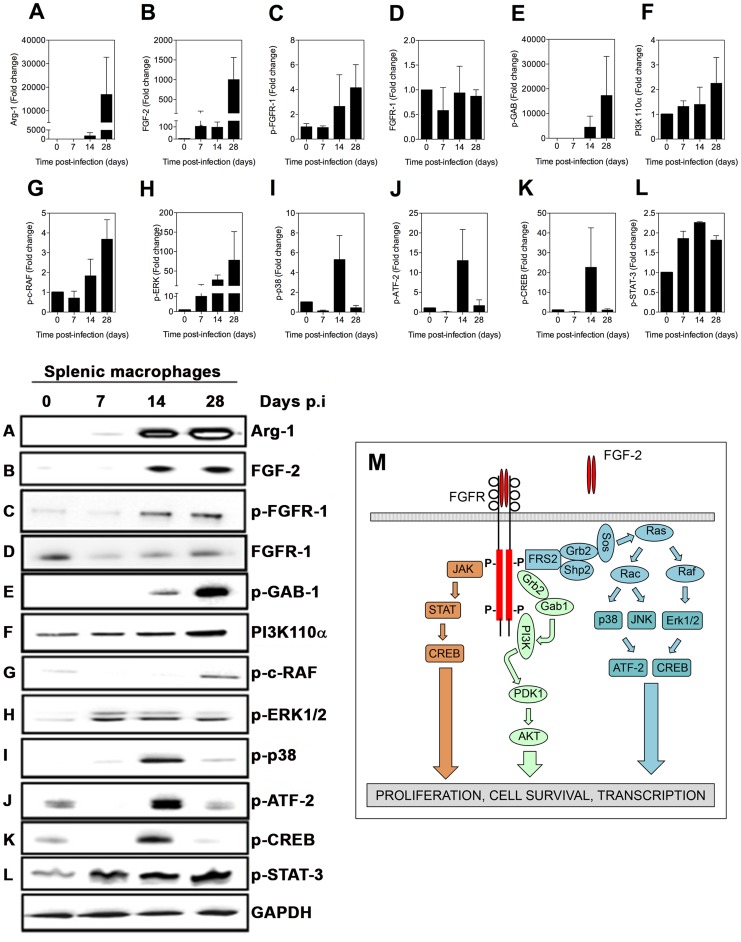
Activation of signaling proteins in the FGFR canonical pathway in splenic macrophages from hamsters with VL. (**A–L**) Splenic macrophages were isolated by adherence from the spleens of uninfected hamsters (time 0) or hamsters infected for 7, 14, and 28 days and whole cell lysates probed with antibodies directed against arg1 (panel A, representative blot A) or members of the FGF signaling pathway (panels and representative blots B–L). Bars represent the fold change with reference to control cells of uninfected hamsters calculated by densitometry analysis of immunoblot bands from samples pooled from 1–4 hamsters per determination from 2–3 independent experiments. **M**) Simplified schematic of the canonical FGF signaling pathway for reference.

**Figure 3:**
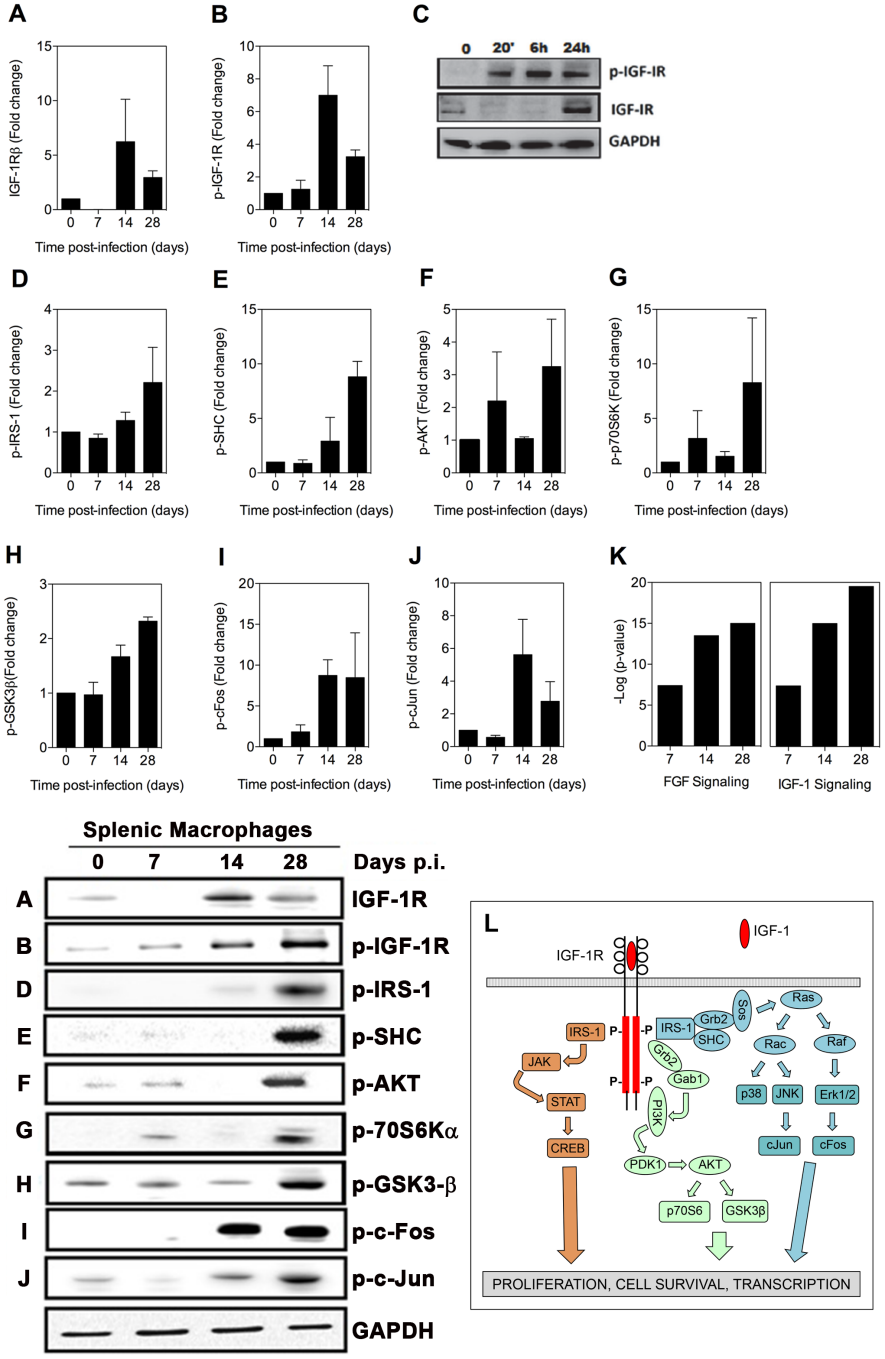
Activation of signaling proteins in the IGF-1R canonical pathway in splenic macrophages from hamsters with VL. (**A–B, D–J**) Immunoblot analysis of expression of proteins in the IGF-1R canonical signaling pathway in splenic macrophages was performed as described in Fig. 2. **C**) Detection of phospho-IGFR by immunoblot in BMDMs uninfected (Un) or infected *in vitro* with *L. donovani* for 20 min to 24 hrs. Shown is an immunoblot from a single experiment. **K**) Network analysis showing the activation of the FGF and IGF-1 canonical signaling pathways generated by comparing the fold change of 32 signaling proteins in splenic macrophages from uninfected and infected (7, 14, and 28 days) hamsters using Ingenuity Pathway Analysis software (Ingenuity Systems). The –log of the *p* value (vertical axis) represents the probability that the association of the data set in that pathway is due to chance. **L**) Simplified schematic of the canonical IGF-1 signaling pathway for reference.

Analysis of the FGF and IGF-1 signaling pathways in splenic macrophages from hamsters with VL by immunoblotting confirmed the finding of the antibody screening array (Figs. 2 and 3). There was no evidence for activation of other growth factor signaling pathways in VL (see Fig. S1 and S2). Our finding that inhibition of EGFR reduced arg1 mRNA expression (Table 1), when neither increased ligand expression nor receptor activation could be demonstrated, suggested that basal activity of EGF/EGFR modulated arg1 expression through an effect on downstream signaling. As we demonstrated previously [2], arg1 protein expression was increased in macrophages isolated from the spleens of hamsters with VL starting at 14 days post-infection (Fig. 2A). Of the growth factor receptor ligands, only FGF-2 expression was increased in splenic macrophages (Fig. 2B, Fig. S1, and Fig. S2) and it was accompanied by increased phosphorylation of Tyr^653/654^ of the FGFR-1 (Fig. 2C) relative to overall receptor protein expression (Fig. 2D). The increase in both FGF-2 and its phosphorylated receptor paralleled the expression of arg1 in the splenic macrophages. Multiple molecules involved in the signaling cascade downstream of FGFR (shown in the diagram in Fig. 2M) were activated, including members of the PI3K/AKT pathway [GAB (Fig. 2E), PI3K (Fig. 2F)] and the MAPK/ERK pathway [c-RAF (Fig. 2G), ERK1/2 (Fig. 2H)]. Activation of p38 MAPK (Fig. 2I), that leads to activation of the transcription factor ATF-2 (Fig. 2J) and the cyclic AMP response element-binding protein (CREB) (Fig. 2K) was observed at 14 days post-infection but was then down-modulated at 28 days post-infection. This suggested that sustained activation of these signaling molecules was not required for the expression of arg1 throughout the course of VL (Fig. 2A). The mechanism(s) through which these molecules are down regulated is unknown. Activation of STAT3, which was evident throughout the course of VL (Fig. 2L), may be a consequence of increased IL-10 production (Fig. S4A and reference [2]) or growth factor signaling (Fig. S4D) [39].

### The IGF-1 signaling pathway is activated in the spleens of hamsters with VL

We were unable to detect increased expression of IGF-1 or IGF-2 in the spleen or plasma of hamsters with VL (Fig. S1; data not shown). However, by immunoblot we found increased expression of the IGF-1R after 14 days post-infection (Fig. 3A), and somewhat unexpectedly the beta (cytoplasmic) domain of the IGF-1 receptor, which mediates intracellular signaling, was phosphorylated at these time points (Fig. 3B). We confirmed these findings in BMDM exposed *in vitro* to *L. donovani* where parasite-induced IGF-1R phosphorylation was evident between 20 minutes and 24 hrs of exposure, and enhanced expression of IGF-1R protein was present at 24 hrs after infection (Fig. 3C). A number of the activated signaling molecules downstream of FGFR overlap with the canonical IGF-1R signaling pathway (compare data in Fig. 2 with schematic in Fig. 3L). Additionally, other pathway members, including IRS-1 (Fig. 3D), SHC (Fig. 3E), AKT (Fig. 3F), p70S6K (Fig. 3G), and GSK3β (Fig. 3H) were activated, as were the downstream transcription factors c-FOS (Fig. 3I) and c-Jun (Fig. 3J). When all of the activated signaling molecules were subjected to network analysis (Ingenuity Pathway Analysis) both the FGFR and IGF-1R pathways were found to be significantly upregulated in splenic macrophages during the course of VL (*p*<10^−7^; Fig. 3K).

### Inhibition of the FGFR and IGFR-1 and downstream signaling molecules decreases arg1 expression and parasite burden in infected macrophages

**Figure 4:**
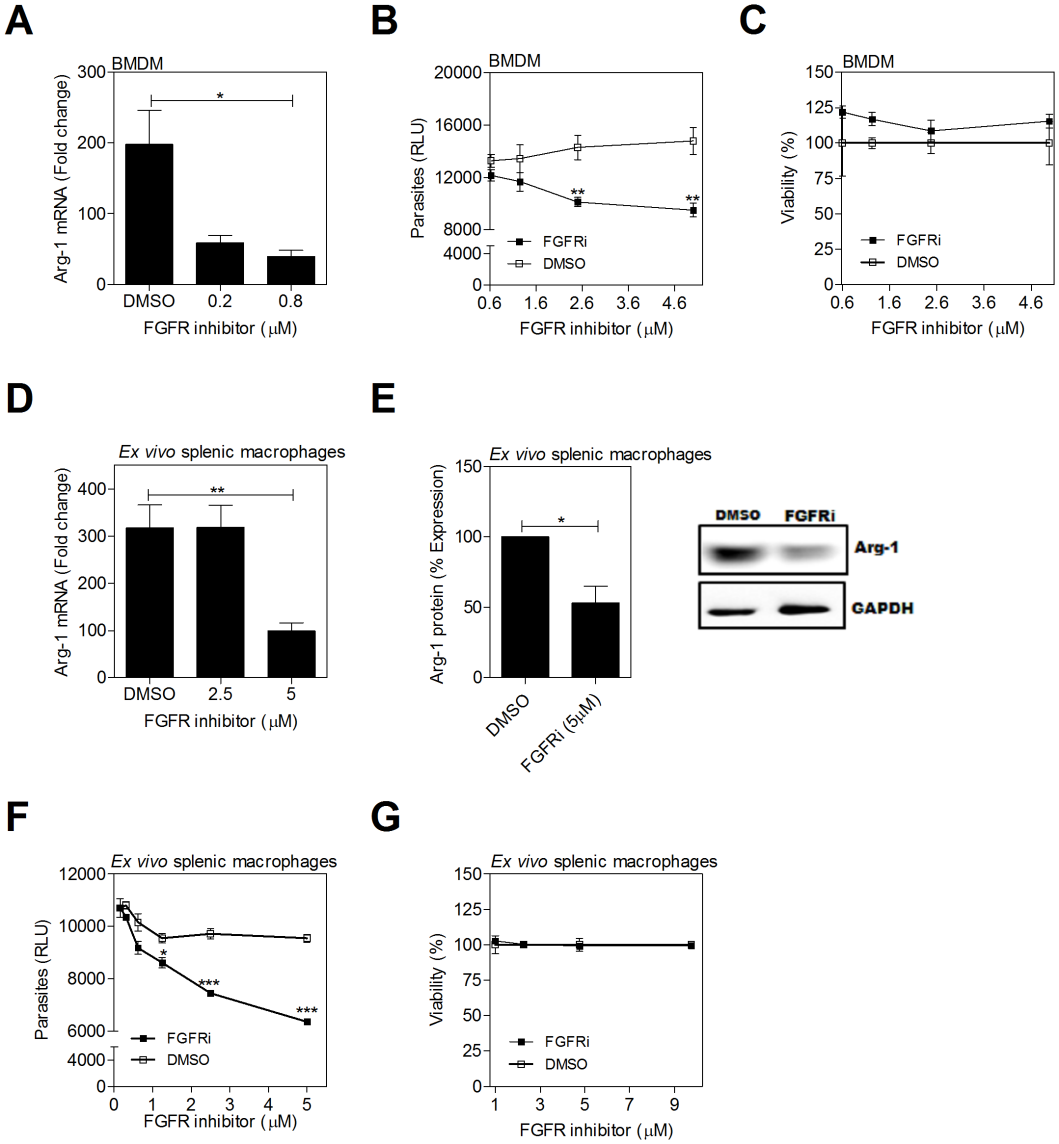
Inhibition of FGFR signaling decreases arg1 expression and parasite burden in *L. donovani* infected macrophages. (**A–C**) Hamster BMDMs were treated with a FGFR-1 inhibitor (PD 166866) or an equivalent concentration of vehicle control (DMSO) and infected *in vitro* with *L. donovani* for 24 or 48 hrs. **A**) arg1 mRNA expression determined by qRT-PCR at 24 hrs post-treatment. **B**) Intracellular parasite burden determined by luminometry from luciferase-transfected *L. donovani* at 48 hrs post-treatment. **C**) Viability of BMDMs determined by luminometry (Cell titer Glo) at 48 hrs post-treatment. **D–G**) Splenic macrophages from *L. donovani* infected hamsters (21–28 days p.i.) were isolated by adherence and cultured *ex vivo* with an inhibitor of FGFR-1 (PD 166866) or an equivalent concentration of vehicle control (DMSO) for 24 or 48 hrs. **D**) arg1 mRNA expression determined by qRT-PCR at 24 hrs post-treatment. **E**) arg1 protein expression determined at 48 hrs post-treatment. Bars represent the percent of expression with reference to control (DMSO treated) cells calculated by densitometry analysis of immunoblot bands from 3 independent experiments. A representative immunoblot is also shown. **F**) Intracellular parasite burden determined by luminometry from luciferase-transfected *L. donovani* at 48 hrs post-treatment. **G**) Viability of splenic macrophages determined by luminometry (Cell titer Glo) at 48 hrs post-treatment. Shown is the mean and SEM of from a single experiment that was representative of 2–4 independent experiments. *p<0.05; **p<0.01.

**Figure 5:**
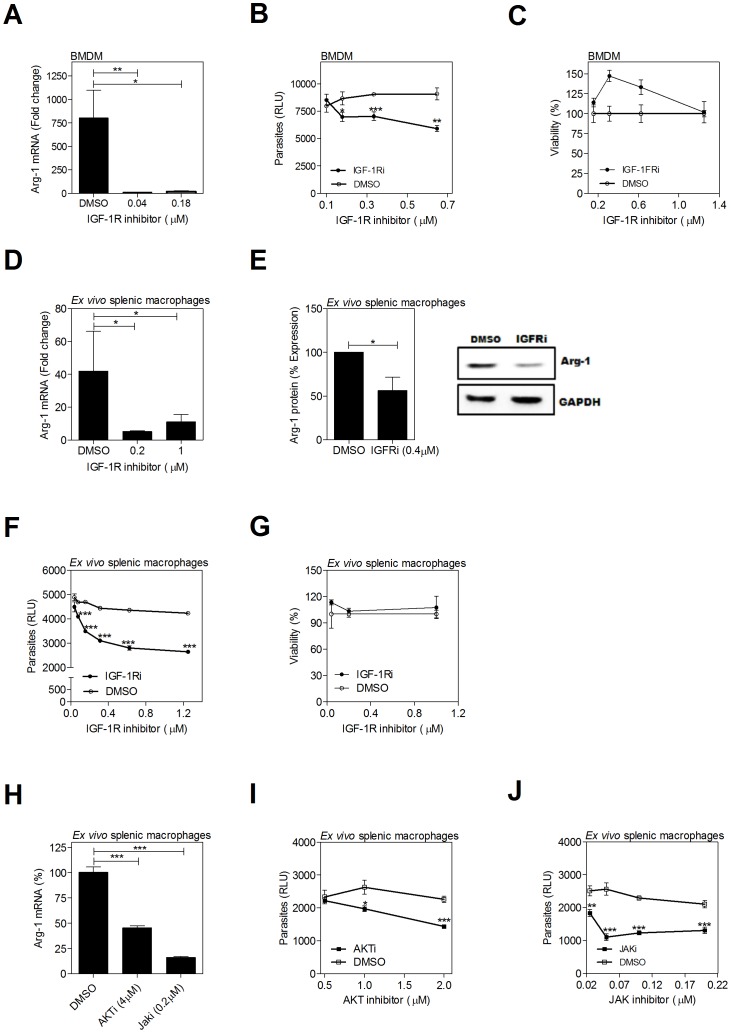
Inhibition of IGF-1R signaling decreases arg1 expression and parasite burden in *L. donovani* infected macrophages. (**A–C**) Hamster BMDMs were treated with an IGF-1R inhibitor (Picropodophyllin, PPP) or an equivalent concentration of vehicle (DMSO) and infected *in vitro* with *L. donovani* for 24 or 48 hrs. (**A**) arg1 mRNA expression determined by qRT-PCR at 24 hrs post-treatment. **B**) Intracellular parasite burden determined by luminometry from luciferase-transfected *L. donovani* at 48 hrs post-treatment. **C**) Viability of BMDMs determined by luminometry (Cell titer Glo) at 48 hrs post-treatment. **D–J**) Splenic macrophages from *L. donovani* infected hamsters (21–28 days p.i.) were isolated by adherence and treated with an IGF-1R inhibitor, AKT inhibitor, or JAK inhibitor, or an equivalent concentration of vehicle control (DMSO) for 24 of 48 hrs. **D**) arg1 mRNA expression determined by qRT-PCR at 24 hrs post-treatment. **E**) arg1 protein expression determined at 48 hrs post-treatment. Bars represent the percent of expression with reference to control (DMSO treated) cells calculated by densitometry analysis of immunoblot bands from 3 independent experiments. A representative immunoblot is also shown. **F**) Intracellular parasite burden determined by luminometry from luciferase-transfected *L. donovani* at 48 hrs post-treatment. **G**) Viability of splenic macrophages determined by luminometry (Cell titer Glo) at 48 hrs post-treatment. **H**) arg1 mRNA expression determined by qRT-PCR at 24 hrs post-treatment with AKT inhibitor (AKTi; CAS# 612847-09-3, Calbiochem), JAK inhibitor (JAKi; CAS# 457081-03-07) or DMSO control. **I–J**) Intracellular parasite burden determined by luminometry from luciferase-transfected *L. donovani* at 48 hrs post-treatment with AKT inhibitor (**I**) or JAK inhibitor (**J**), compared to DMSO treated controls. In each of the panels the mean and SEM from a single experiment that was representative of 2–3 independent experiments is shown. *p<0.05; ***p<0.001.

Treatment of *L. donovani*-infected hamster BMDMs over 24 hrs of infection with an inhibitor of FGFR-1 resulted in a significant dose-dependent reduction of arg1 mRNA expression (Fig. 4A) and parasite burden (Fig. 4B) without affecting cell viability (Fig. 4C). Notably the concentration of FGFR inhibitor required to inhibit parasite replication was higher than the concentration that reduced arg1 expression. This suggests that growth factor signaling supported parasite growth/survival through additional arg1-independent mechanisms, or that residual arginase activity at the lower inhibitor concentration is enough to support parasite growth. The latter possibility is consistent with our previous finding that >90% arg1 knockdown led to approximately 50% reduction of parasite load [2]. The FGFR inhibitor also blocked the expression of arg1 mRNA (Fig. 4D) and protein (Fig. 4E), and reduced the parasite burden (Fig. 4F) without affecting cell viability (Fig. 4G) in ex *vivo* cultured spleen cells from infected hamsters. Similar effects were found by inhibition of IGF-1R. In the *in vitro* infection model, IGF-1R inhibition reduced parasite-induced expression of host arg1 mRNA (Fig. 5A) and the intracellular parasite load (Fig. 5B), without decreasing cell viability (Fig. 5C). Similarly, the inhibitor reduced arg1 mRNA (Fig. 5D) and protein (Fig. 5E), and reduced the parasite burden (Fig. 5F) without affecting cell viability (Fig. 5G) in *ex vivo* cultured spleen cells from infected hamsters. The FGFR and IGF-1R inhibitors did not have a direct effect on the viability of *L. donovani* cultured promastigotes (Fig. S3), suggesting that the effect of receptor inhibition was through modulation of the host cell. Inhibition of JAK, which plays a key role in the phosphorylation of STAT proteins following cytokine and growth factor signaling, dramatically reduced arg1 transcription in *ex vivo* cultured splenocytes from infected hamsters (Fig. 5H). To a lesser degree, inhibition of the protein AKT, which is involved in signal transduction downstream of the IGF-1 and FGF receptors, also decreased Arg-1 expression (Fig. 5H). Both the AKT and JAK inhibitors significantly reduced parasite load (Figs. 5I and 5J).

### Cytokines amplify the *L. donovani*- and growth factor-induced expression of arginase 1

**Figure 6:**
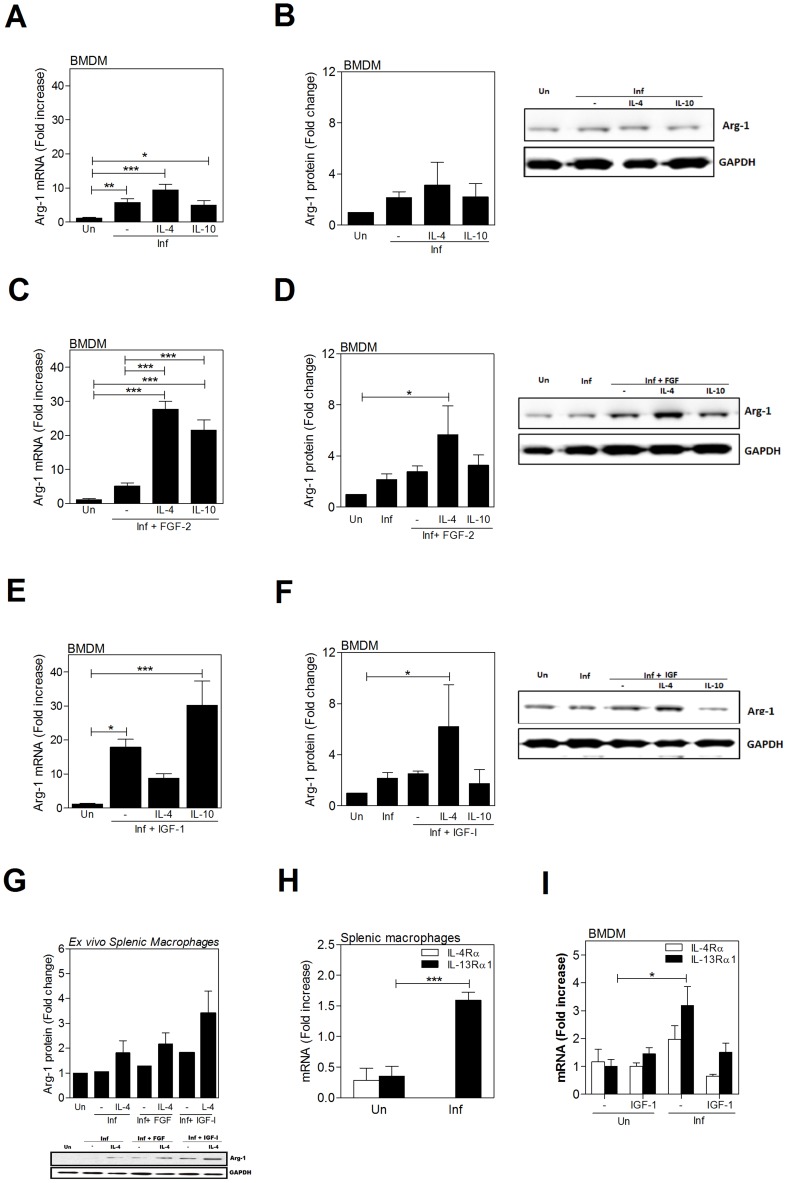
IL-4 enhances growth factor-induced arg1 in *L. donovani* infected macrophages. Infected hamster BMDM were exposed or not to hamster IL-4 (25 IU/mL), recombinant human IL-10 (100 ng/mL), recombinant human FGF-2 (20 ng/mL) and/or recombinant human IGF-1 (100 ng/mL) for 24 or 48 hrs. **A, C, E**) Arg1 mRNA expression determined by qRT-PCR at 24 hrs post-treatment. Shown is the mean and SEM of 6 replicates from a single experiment that was representative of 2 independent experiments. **B, D, F**) Arg1 protein expression determined in *L. donovani* infected BMDM exposed to IL-4, IL-10, and growth factors, alone or in combination, for 48 hrs. The membranes were stripped and stained with antibody against GAPDH to confirm equivalent protein loading. Bars represent the fold change with reference to control cells of uninfected hamsters calculated by densitometry analysis of immunoblot bands from 3 independent experiments. Also shown is a representative individual immunoblot. **G**) Arg1 protein expression determined in splenic macrophages from uninfected and *L. donovani* infected hamsters exposed *ex vivo* to IL-4 and growth factors, alone or in combination, for 48 hrs. The membranes were stripped and stained with antibody against GAPDH to confirm equivalent protein loading. Bars represent the fold change with reference to control cells of uninfected hamsters calculated by densitometry analysis of immunoblot bands from 3 independent experiments. Also shown is a representative individual immunoblot. **H**) Expression of IL-13Rα1 and IL-4Rα mRNA in splenic macrophages from uninfected (0) or 18-day infected hamsters determined by qRT-PCR. **I**) Expression of IL-13Rα1 and IL-4Rα mRNA in BMDM from uninfected (Un) and *L. donovani* infected (Inf) BMDMs (24 hrs p.i.) stimulated or not with IGF-1 or FGF-2. Shown is mean and SEM of the fold increase of receptor expression over uninfected, unstimulated controls from a single experiment representative of 2 independent experiments. *p<0.05; **p<0.01; ***p<0.001.

Since cytokines (IL-4 and IL-10) are known to stimulate the expression of arginase [5], [6], and we demonstrated that growth factors also induced arginase (Fig. 1), we investigated the potential for amplification of arg1 expression in macrophages by simultaneous exposure to these stimuli (all of which are expressed in the spleen during VL (reference [2] and Figs. 2 and 3). The *L. donovani*-induced expression of arg1 in BMDM was modestly amplified by IL-4 but not IL-10 at the mRNA level (Fig. 6A), but neither significantly amplified the arg1 protein (Fig. 6B). However, IL-4 and IL-10 dramatically enhanced the FGF-2-induced arg1 mRNA (Fig. 6C), and IL-4 (but not IL-10) enhanced FGF-2-induced arg1 protein (Fig. 6D) expression in infected macrophages. IL-4 did not amplify IGF-1-induced arg1 mRNA expression in infected BMDMs (Fig. 6E) but augmented arg1 protein expression (Fig. 6F). Similar to IL-10 and FGF-2, IL-10 enhanced IGF-1-induced arg1 mRNA but not protein expression. A trend of an additive effect of IL-4 and the growth factors was also found in splenic macrophages from infected animals exposed to the cytokine and growth factors *ex vivo* (Fig. 6G). The additive effect of IL-4 and growth factors in the induction of arg1 expression prompted us to consider that there may be cross-regulation of receptor expression. We found that the expression of IL-13Rα1, but not IL-4Rα, was upregulated in splenic macrophage from hamsters with VL (Fig. 6H) and in BMDMs infected with *L. donovani* (Fig. 6I). Addition of FGF-2 or IGF-1 to infected macrophages did not further increase the expression of either of these receptor components (data not shown and Fig. 6I). IL-10Rα expression (along with IL-10) was also increased in splenic macrophages from infected hamsters (Fig. S4A) and in *in vitro* infected BMDMs (Fig. S4B), but FGF-2 or IGF-1 did not augment IL-10 or IL-10Rα expression (Fig. S4B). These data, coupled with the data shown in Figs. 2 and 3, suggest that the cytokine-mediated amplification of growth factor driven arg1 could occur by either increased IL-4-mediated signaling through upregulated type II receptor (IL-13Rα1) expression [40] or through activation of signaling proteins (e.g. Jak-1, STAT6, IRS-1, PI3K, AKT) common to the two pathways.

### STAT6 is required for *L. donovani* induced expression of arg1 in macrophages

**Figure 7:**
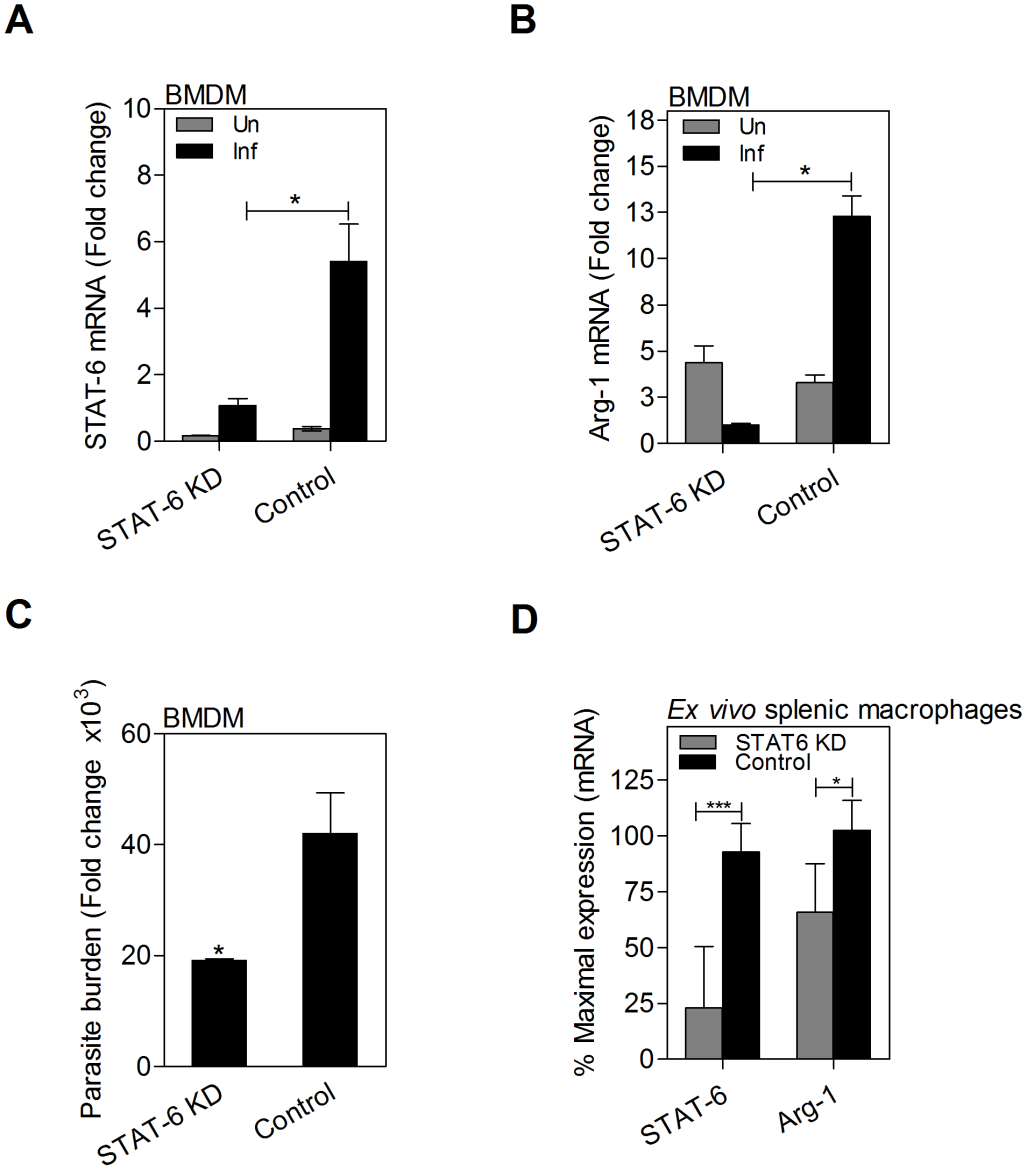
Parasite-induced arg1 expression in macrophages is dependent on STAT6. Expression of **A**) STAT6 mRNA and **B**) arg1 mRNA in BMDMs that were uninfected (Un) or infected *in vitro* with *L. donovani* (Inf) for 24 h after transfection with STAT6-specific knockdown siRNA (STAT6 KD) or scrambled siRNA (Control). Shown is the mean and SEM of the fold-change in mRNA compared to unstimulated controls as determined by qRT-PCR in 6 replicates from 2 independent experiments. **C**) Parasite burden at 24 h post-infection of STAT6 KD BMDMs or control. Shown is the mean and SEM of the parasite burden with reference to control (uninfected) cells in 4 replicates determined by qRT-PCR. **D**) STAT6 and arg1 mRNA expression in splenic macrophages from *L. donovani* infected hamsters 48 hrs after *ex vivo* transfection with STAT6-specific siRNA (STAT6 KD) or scrambled siRNA (Control). Data are shown as the mean and SEM of the percent of maximal mRNA expression in the control samples. *p<0.05; ***p<0.001.

**Figure 8:**
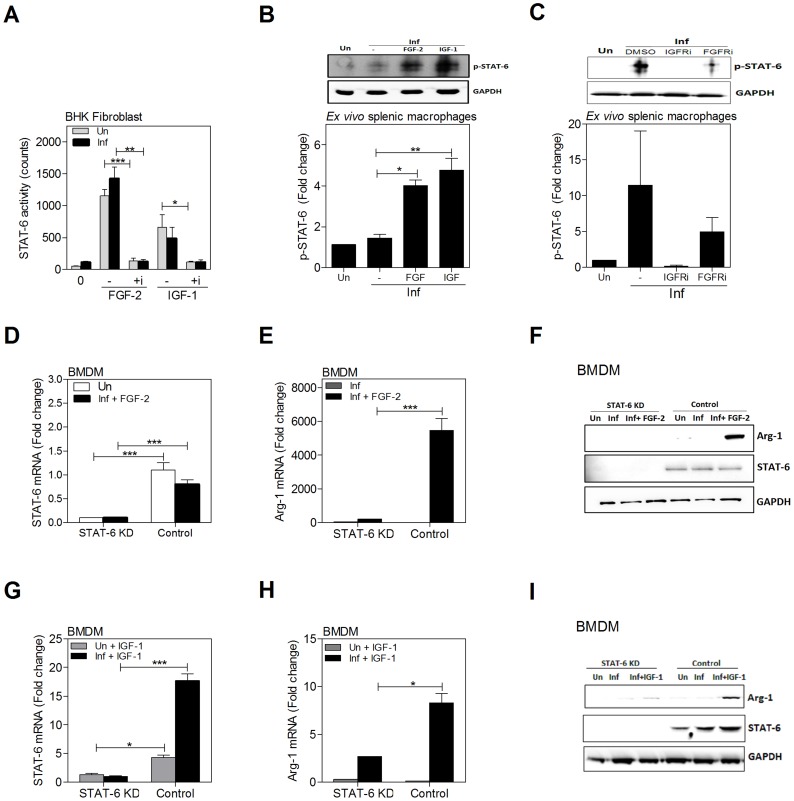
Growth factors and cytokines converge at STAT6 to induce arg1 expression in *L. donovani* infected macrophages. **A**) Growth factors activate STAT6. Hamster BHK fibroblasts transfected with a STAT6 luciferase reporter were uninfected (Un) or infected (Inf) with *L. donovani* and stimulated for 24 h with FGF-2 (20 ng/mL) or IGF-1 (100 ng/mL) in the absence or presence (+i) of 250 nM of an inhibitor of FGFR-1 (PD166866) or IGF-1R (PPP). Shown is the mean and SEM of luciferase activity from 3 replicates from a single experiment that was representative of 2 independent experiments. **B**) phospho-STAT6 expression in BMDMs stimulated with IGF-1 and FGF-2. BMDMs were uninfected (Un) or infected *in vitro* with *L. donovani* (Inf) and stimulated or not with FGF-2 (20 ng/mL) or IGF-1 (100 ng/mL) for 20 min. STAT6 protein was immunoprecipitated in cell lysates and phosphorylated STAT6 determined by immunoblot. GAPDH was used to confirm that equivalent amounts of protein were subjected to the immunoprecipitation. Bars represent the mean and SEM of fold change with reference to the uninfected controls calculated by densitometry analysis of immunoblot bands from 6 independent experiments. Also shown is a representative individual immunoblot. **C**) Blockade of STAT6 activation by IGF-1R and FGFR inhibitors. Splenic macrophages from uninfected hamsters were pre-treated with IGF-1R inhibitor (100 nM PPP) or FGFR inhibitor (300 nM PD166866) and infected *in vitro* with *L. donovani* for 20 min in absence of the inhibitor. STAT6 protein was immunoprecipitated in cell lysates and the level of phosphorylated STAT6 determined by immunoblot. Data shown is from 3 independent experiments, with a representative individual immunoblot, as described for Fig. 8B. **D**) Expression of STAT6 mRNA in BMDMs that were uninfected-unstimulated (Un), infected (Inf), or infected and stimulated with FGF-2 after transfection with STAT6-specific siRNA (STAT6 KD) or scrambled siRNA (Control). Shown is the mean and SEM of the fold-increase in STAT6 mRNA with reference to uninfected control as determined by qRT-PCR in 4–10 replicates from a single experiment that was representative of 3 independent experiments. **E**) Abrogation of arg1 mRNA expression by knockdown of STAT6 in BMDMs infected *in vitro* with *L. donovani* and stimulated with FGF-2. BMDMs were transfected with the siRNA as described above and then infected and stimulated with FGF-2 (20 ng/mL) for 24 hrs. The data are shown as the mean and SEM of the fold-increase in arg1 mRNA relative to negative (uninfected) control cells from a single experiment that was representative of 3 independent experiments. **F**) Immunoblot showing efficiency of siRNAi-mediated knockdown of STAT6 protein in hamster BMDMs and the requirement of STAT6 in the FGF-2-induced arg1 expression in *L. donovani* infected cells. Following transfection with the STAT6-specific (STAT6 KD) or control siRNAi the BMDMs were uninfected (Un), infected with *L. donovani* (Inf), or infected and treated with FGF-2 (20 ng/mL) for 48 hrs. **G**) Expression of STAT6 mRNA in BMDMs that were uninfected and stimulated with IGF-1 (200 ng/mL) or infected (Inf) and stimulated with IGF-1 after transfection with STAT6-specific siRNA (STAT6 KD) or scrambled siRNA (Control). Shown is the mean and SEM of the fold-increase in STAT6 mRNA compared to IGF-1-treated STAT6 KD cells as determined by qRT-PCR in 3 replicates from a single experiment that was representative of 2 independent experiments. **H**) Abrogation of arg1 mRNA expression by knockdown of STAT6 in BMDMs infected *in vitro* with *L. donovani* and stimulated with IGF-1. BMDMs were transfected with the siRNA as described above and then infected and stimulated with IGF-1 (200 ng/mL) for 24 hrs. The data are shown as the mean and SEM of the fold-increase in arg1 mRNA as determined by qRT-PCR relative to nonstimulated cells from a single experiment that was representative of 2 independent experiments. **I**) Immunoblot showing efficiency of siRNAi-mediated knockdown of STAT6 protein in hamster BMDMs and the partial requirement of STAT6 in the IGF-1-induced arg1 expression in *L. donovani* infected cells. Experiment was designed and data presented as described for Fig. 8F. IGF-1 was used at 200 ng/mL. Shown is an immunoblot from a single experiment that was representative of 2 independent experiments. *p<0.05; **p<0.01; ***p<0.001.

We previously demonstrated that STAT6 was required for *L. donovani*-induced arg1 expression in fibroblasts [2]. Here we confirmed that siRNA-mediated knockdown of STAT6 mRNA (Fig. 7A) and protein (see Fig. 8F and 8I) in *in vitro* infected macrophages led to reduced arg1 mRNA (Fig. 7B) expression, and improved control of parasite replication (Fig. 7C). Similarly, knockdown of STAT6 (75% reduction) in *ex vivo* cultured splenic macrophages from infected hamsters led to significantly reduced arg1 mRNA expression (Fig. 7D). These data confirm the critical importance of STAT6 in the parasite-driven expression of arg1 in macrophages in VL.

### Growth factors activate STAT6 and increase STAT6-dependent arg1 expression

Since STAT6 had a critical role in parasite-induced arg1 transcription, activation of growth factor signaling was evident in *L. donovani* infection, and there was an additive effect of IL-4 and growth factors in the induction of arg1 expression, we wanted to know if the FGF-2- and/or IGF-1-induced arg1 expression was dependent on the activation of STAT6. In a STAT6 reporter assay (hamster fibroblast cell line; reference [2]), we found that recombinant FGF-2 and IGF-1 induced STAT6 activation, which was blocked when cells were pre-treated with an inhibitor of the corresponding growth factor receptor (Figure 8A). In the fibroblast cell line, exposure to parasites had a relatively weak effect on STAT6 activation, probably because at this parasite dose the cells are infected at a very low level. The growth factor-induced activation of STAT6 in macrophages was confirmed by detection of phosphorylated STAT6 in immunoprecipitated lysates of splenic macrophages from *L. donovani* infected hamsters exposed *ex vivo* to recombinant FGF-2 or IGF-1 (Fig. 8B). Parasite-induced STAT6 activation was abrogated completely by an IGF-1R inhibitor and partially by an FGFR inhibitor (Fig. 8C). Conversely, siRNA-mediated knockdown of STAT6 mRNA in infected, FGF-2-treated BMDM (Fig. 8D) identified the requirement for STAT6 in the FGF-2-induced expression of arg1 mRNA (Fig. 8E) and protein (Fig. 8F). Similarly, siRNA-mediated knockdown of STAT6 in infected IGF-1-treated BMDM (Fig. 8G) identified the contribution of, but not absolute requirement for, STAT6 in the IGF-1-induced expression of arg1 mRNA (Fig. 8H) and protein (Fig. 8I). Collectively these data identify the critical importance of growth factor signaling in the parasite-induced activation of STAT6, and of STAT6 in the IGF-1 and FGF-2 driven expression of arg1 in *L. donovani* infected macrophages.

### IL-4 and growth factors have an additive effect in the activation of STAT6

**Figure 9:**
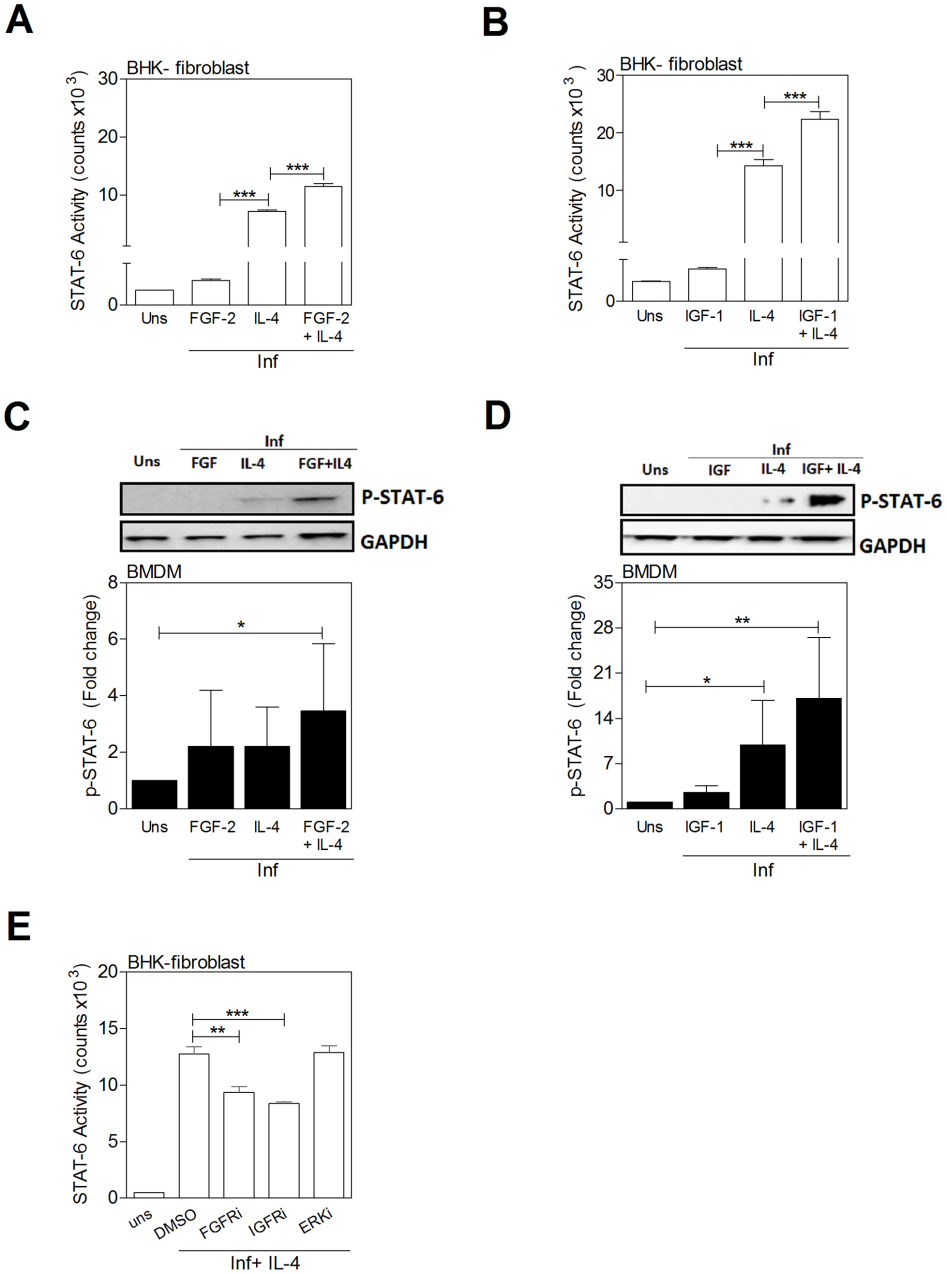
IL-4 and growth factors amplify STAT6 activation. **A, B**) STAT6 activation measured in a luciferase reporter assay in BHK cells stimulated for 24 h with growth factors and a sub-maximal concentration of IL-4. **A**) IL-4 (3 IU/mL) and/or FGF-2 (20 ng/mL) and **B**) IL-4 (6 IU/mL) and/or IGF-1 (100 ng/mL). Shown is the mean and SEM of STAT6 activity determined by luminometry from a single experiment that was representative of 3 independent experiments. **C, D**) Immunoblots of cell lysates (not immunoprecipitation as shown in Fig. 8B) showing phospho-STAT6 in BMDMs stimulated for 20 min with *L. donovani* promastigotes and **C**) IL-4 (8 IU/mL) and/or FGF-2 (20 ng/mL) or **D**) IL-4 (20 IU/mL) and/or IGF-1 (100 ng/mL). Bars represent the mean and SEM of fold change with reference to the uninfected, unstimulated (Uns) controls calculated by densitometry analysis of immunoblot bands from 3 independent experiments. Also shown is a representative individual immunoblot. **E**) IL-4-mediated activation of STAT6 in infected BHK fibroblasts is reduced by inhibition of FGFR and IGF-1R but not ERK. The cells were exposed to the control (DMSO) or FGFR inhibitor (FGFRi; PD166866; 10 µM), IGFR inhibitor (IGFRi; PPP; 5 µM), or ERK inhibitor (ERKi; PD98059; 5 µM) for 1 hr and then stimulated for another 24 hrs with IL-4 (25 IU/mL) in the presence or absence of inhibitor. Shown is the mean and SEM of STAT6 activity determined by luminometry from a single experiment that was representative of 2 independent experiments.

Since simultaneous exposure of infected macrophages to IL-4 and FGF-2 or IGF-1 led to enhanced arginase expression, and the growth factor- and cytokine-induced expression of arg1 was dependent on STAT6, we reasoned that there might be enhanced activation of STAT6 in cells exposed to both IL-4 and growth factors. Stimulation of the reporter cells with either growth factors (see also Fig. 8A) or IL-4 activated STAT6. There was evidence of an additive effect when the growth factor and cytokine were combined (Figs. 9A and 9B). By immunoblotting, STAT6 phosphorylation was amplified when IL-4 was combined with the growth factors (Figs. 9C and 9D). Inhibition of FGFR and IGF-1R activation led to decreased IL-4-induced STAT6 activation (Fig. 9E). Taken together, these data indicate that bi-directional crosstalk between the growth factor and IL-4 signaling pathways converges at STAT6 to drive arg1 expression in VL.

## Discussion

In an experimental model of progressive VL, we demonstrated previously that parasitized macrophages were polarized to an M2-like phenotype [2], characteristic of macrophages at a site of chronic injury and wound healing [5], [6], and were massively expanded in the spleen [2], [38]. These macrophages had dominant expression of arg1, which promoted parasite growth. The *L. donovani*-induced macrophage arg1 expression did not require, but was amplified by, type 2 cytokines [2]. In this work we focused our attention on the mechanisms through which pathological arg1 expression occurs in VL. We discovered that FGF-2 and IGF-1 signaling pathways were activated in splenic macrophages from animals with progressive VL. These growth factors, which may be produced by macrophages, fibroblasts, or endothelial cells [41]–[43], induced macrophage arg1 expression. Inhibition of FGFR1 and IGF-1R signaling led to both reduced arg1 expression and improved control of intracellular *L. donovani* infection. Parasite-induced FGFR and IGF-1R signaling converged with the canonical type 2 cytokine signaling pathway through STAT6 activation to induce arg1 expression. Simultaneous exposure of macrophages to growth factors and IL-4, as would occur in the spleen during VL, enhanced the activation of STAT6 and expression of arg1. The interplay of STAT6 and growth factor signaling was confirmed by demonstrating that FGF-2- and IGF-1-induced arg1 expression was abrogated by knockdown of STAT6, and conversely, that inhibition of growth factor signaling reduced parasite- and IL-4-mediated STAT6 activation and arg1 expression.

Arginase expression contributes to the pathogenesis of cutaneous *L. major* infection in mice [19]–[23] and progressive experimental VL caused by *L. donovani* [2]. Its expression in blood leukocytes was also found to be a marker of active VL in patients from Ethiopia [35]. In that study the blood leukocytes that produced arginase were found in the mononuclear cell fraction but expressed CD15 so were identified as low-density granulocytes. Those cells were not further characterized, and we have not evaluated expression of arg1 in granulocytes in our model of experimental VL. Therefore, it remains to be determined if there is a fundamental difference in the source of arg1 in experimental and human VL, or if further characterization of the cell populations will resolve the apparent difference. The disease-promoting effect of arg1 may be mediated through several mechanisms. First, arg1 metabolizes arginine such that this substrate is not available for the generation of the antimicrobial effector molecule, nitric oxide, by the action of inducible nitric oxide synthase. Second, arg1 expression leads to the production of polyamines, which promote intracellular *Leishmania* growth [2], [19], [20]. Lastly, local depletion of arginine leads to impaired anti-leishmanial T cell responses [44]. The relative contributions of each of these effects on the pathogenesis of VL remain to be determined.

The role of growth factors in modulation of arg1 expression and macrophage function in response to *Leishmania* or other pathogens has received little attention. The induction of arginase expression is classically a type 2 cytokine (IL-4/IL-13)- and STAT6-driven process [5], although some parasites or parasite products have been shown to directly induce an M2-like macrophage phenotype [2], [20], [45]. Since growth factors modulate inflammation and tissue repair [46]–[50], processes in which M2 macrophages have an integral part, it is not surprising that there would be interconnections between growth factors, type 2 cytokines, and M2 polarization. The tissue remodeling [51], [52], accumulation of macrophages [38], [52]–[55] and collagen deposition/fibrosis [38], [54] observed in the spleens in experimental and human VL are processes that suggest growth factors may contribute to VL pathology. Cytosolic IGF-1 was found increased in *L. major* infected murine macrophages [56], and IGF-1 induced parasite arginase in *L. amazonensis* infected macrophages [57]. Although we cannot exclude the potential contribution of parasite arginase in the IGF-1 and FGF-2-mediated effects on macrophages, we found previously that *L. donovani* arginase contributed little to the overall arginase expression at the site of infection in this model of progressive VL [2]. The increased expression of FGF-2 and evidence of signaling through the IGF-1 and FGF receptors to our knowledge had not been described previously in VL. Surprisingly, robust IGF-1R phosphorylation was evident in the infected spleen in the absence of increased IGF-1, suggesting cross-activation by FGF-2 [58] or by an unknown host or parasite-derived factor. We think cross-activation by FGF-2 is unlikely in the case of VL since we did not find IGF-1R phosphorylation in BMDMs infected with *L. donovani* and treated with FGF-2 for 20 minutes to 48 hours post-infection (data not shown). Of note, it was reported previously that *Leishmania* expressed an ortholog of FGF-2 [59] so conceivably other parasite-produced growth factor orthologs could be driving the activation of IGF-1R in the absence of host IGF-1. Insulin-like growth factor binding proteins (IGFBPs) or IGFBP proteases [60] could also be modulating the local availability and activity of IGF-1 during the infection.

From this work we have begun to understand the mechanistic basis for the interplay of *L. donovani*, IL-4 and growth factors in the induction of arg1. IL-4, which is increased in the spleen during VL in humans and hamsters [2], [12], amplifies the parasite- and growth factor-induced expression of arg1. IL-10 appears to have a more limited role in that it upregulates arg1 mRNA, but not protein expression, in infected macrophages, and does not amplify the growth factor effect. Similarly, in *L. donovani*-infected mice, IL-10 does not directly induce macrophage arginase, but contributes indirectly to its expression by upregulating the type I IL-4 receptor [61]. FGF-2- and IGF-1 enhance expression of arg1 in *L. donovani* infected macrophages, but have a more modest effect on uninfected macrophages. Thus, the concomitant expression of IL-4 and growth factors in the infected spleen provide an environment highly suited for arg1 expression. IL-4 and IL-13 were shown previously to induce the expression of macrophage IGF-1 [62] and coincident expression of type 2 cytokines and IGF-1 was demonstrated in experimental helminth infection [63]. The amplification of growth factor-induced arg1 by IL-4 in experimental VL is not associated with growth factor-mediated upregulation of the type 1 IL-4 receptor (IL-4rα). Although we found that *L. donovani* infection increased expression of IL-13Rα1, which partners with IL-4rα to transduce a signal via IL-4 or IL-13 [40], this receptor is thought to be a less-potent driver of M2 macrophage activation than is IL-4 signaling through the type I receptor. Furthermore, IRS-1/2, which we found strongly activated in VL, is activated primarily via IL-4 signaling through the type I rather than the type II receptor [64], [65]. Collectively, these data suggest that the growth factor/IL-4-mediated amplification of arg1 expression results from an effect downstream of the IL-4 receptor. Since IL-13 [2] and its receptor are also increased in the spleen during VL, they may also contribute to the induction of arg1.

**Figure 10:**
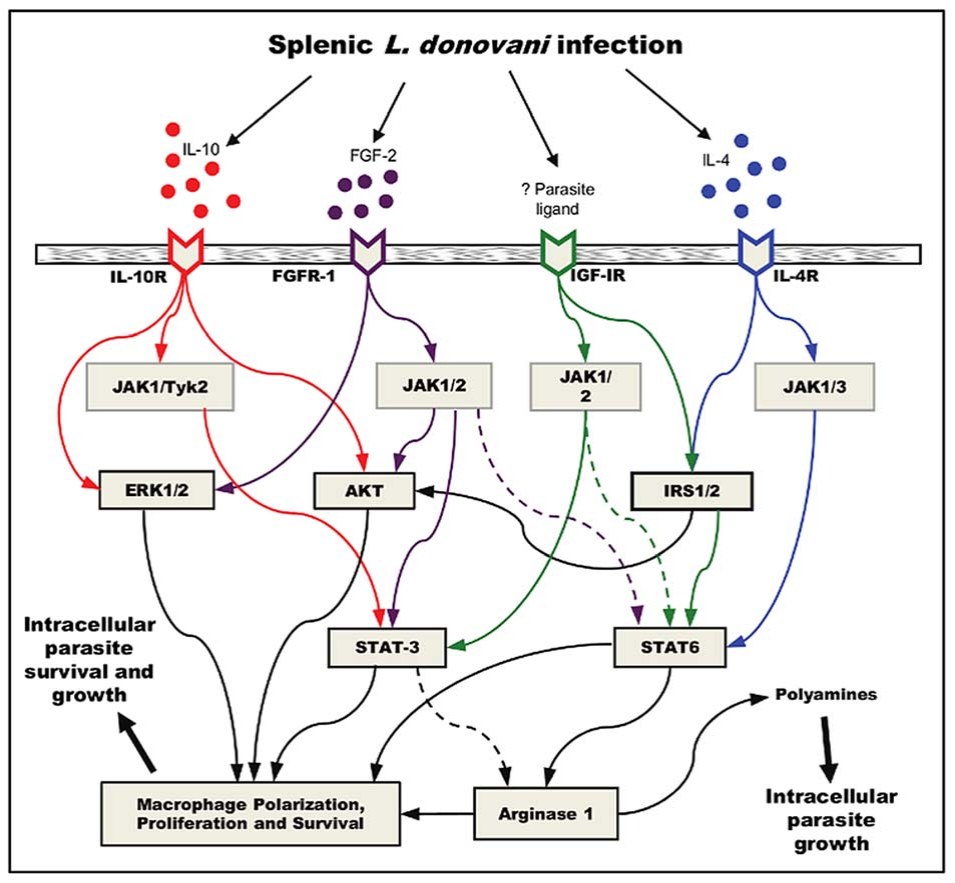
Working model for convergent signaling of growth factors and cytokines in the induction of arg1 in VL. *L. donovani* infection induces the production of IL-4, IL-10 and FGF-2 in the spleen. FGFR is activated, as is IGF-IR by a yet to be identified host or parasite ligand. JAK kinases are phosphorylated through the activated cytokine or growth factor receptors, which lead to IRS-1/2, AKT, ERK, and STAT activation. Translocated STAT6, and possibly STAT3 lead to the transcriptional activation of arginase, which generates polyamines from arginine and leads to parasite growth. These transcription factors also contribute to the polarization macrophages toward an M2 phenotype, which is more permissive to *L. donovani* survival and growth. The collective effect of AKT, ERK, and STAT3 activation, and the generation of polyamines, are likely to lead to growth, proliferation and survival of arginase expressing cells, but this needs experimental confirmation in VL. Solid lines indicate known mechanistic interactions; dashed lines represent suppositional interactions. Only key shared signaling proteins are included in the model.

The body of work presented here supports the conclusion that the signaling pathways downstream of the growth factor and IL-4 receptors converge at STAT6 to drive pathological arg1 expression. Figure 10 illustrates our current working model for the expression of arg1 in VL. IGF-1 is known to activate STAT6 through an IRS-1/2-dependent pathway. IL-4, which is also an activator of IRS-1/2 [64], can amplify this effect [66], [67]. To our knowledge FGF-2 had not been shown previously to activate STAT6. The pathway through which the growth factors activate STAT6-dependent arg1 transcription in VL remains to be fully elucidated, but for the reasons noted above, IRS-1/2 and JAKs, which are activated in splenic macrophages during VL, are likely key intermediates (see Fig. 10). The downstream activation of other transcription factors (CREB, STAT-3) and signaling molecules, including PI3K/AKT, ERK, p38 MAPK, and GSK3β, are also likely to directly or indirectly contribute to the growth factor induced macrophage polarization and arg1 expression. Notably, p38 MAPK and downstream transcription factors (CREB and ATF-2) are only transiently upregulated so do not account for the sustained increase in arg1 expression over time (reference [2] and this work). Down-modulation of the p38 pathway, however, may contribute to the survival and local expansion of splenic arginase-expressing macrophages [68]. *Leishmania* infection of macrophages was shown previously to activate the PI3K/AKT pathway, which is a critical regulator of the IL-10 and IL-12 response [69]–[71]. *L. donovani*-induced production of IL-10 by macrophages involved activation of the PI3K/AKT pathway and downstream phosphorylation-mediated inactivation of GSK-3beta and phosphorylation of CREB [72]. Our data suggest that parasite-induced arg1 is driven at least in part through activation of the same pathway that mediates production of IL-10 by macrophages. However, arg1 and IL-10 expression appear to result from parallel rather than interdependent processes because IL-10 was not a strong inducer of arg1 and did not amplify growth factor-induced arg1 as did IL-4. Also the inhibition of the AKT pathway, which drives IL-10 production, had a less dramatic effect in the down regulation of arg1 than the inhibition of JAKs with the consequent block in STAT activation. Taken together, these data indicate that the expression of arg1 downstream of the growth factor/PI3K/AKT pathway, which is enhanced by IL-4/STAT6 signaling, is an additional mechanism of parasite-mediated subversion of macrophage effector function. Further work is needed to definitively determine the role of IL-10 and STAT3 in this process.

The pathological signaling through the IGF-1R and FGFR that leads to arginase expression in progressive VL is a potential target for adjunctive chemotherapy. Therapies targeting these pathways have recently emerged for a number of proliferative diseases, in particular hematopoietic malignancies and solid tumors (reviewed in [73], [74]). Our *ex vivo* data suggest that inhibition of FGFR or IGF-1R signaling could have therapeutic potential. Furthermore, it was previously demonstrated in a murine model of *L. donovani* infection that *in vivo* administration of a receptor tyrosine kinase inhibitor, when combined with conventional anti-leishmanial chemotherapy had a therapeutic effect [75]. Future pre-clinical studies of FGFR and IGF-1R inhibitors, alone or in combination with current anti-leishmanial therapies, are warranted.

In summary, we determined that the convergence of FGFR/IGFR and IL-4 signaling pathways is responsible for the expression of arg1 in disease-promoting macrophages during chronic progressive VL. FGF-2 and Th2 cytokines [2] are produced in the spleen and lead to activation of the FGFR and STAT6 in infected splenic macrophages. Although the infection does not appear to increase IGF-1 production, the IGF-1R is activated on splenic macrophages through a yet to be identified host or parasite factor. Activation of the FGF and IGF-1 receptors leads to phosphorylation of downstream signaling molecules such as IRS1/2, PI3K, and AKT, which lead to expression of IL-10 [72] and converge with downstream components of the IL-4R pathway to drive arg1 expression. Activation of these pathways, along with the parallel effects of IL-10 in subverting macrophage function [16], [76], [77], plays an important role in the pathogenesis of VL. Targeted interruption of these pathological processes offers an approach to restrain this relentlessly progressive disease.

## Materials and Methods

### Ethics statement

This study was carried out in strict accordance with the recommendations in the Guide for the Care and Use of Laboratory Animals of the National Institutes of Health. The protocol was approved by the Institutional Animal Care and Use Committee of the University of Texas Medical Branch, Galveston, Texas (protocol number 1101004).

### Hamsters

6–8 week old Syrian golden hamsters (*Mesocricetus auratus*) were obtained from Harlan Laboratories.

### Parasites and infection

*L. donovani* (MHOM/SD/001S-2D) promastigotes were cultured as described previously [78]. Hamsters were infected by intracardial injection of 10^6^ peanut agglutinin purified metacyclic promastigotes [78]. For *in vitro* infections, stationary phase promastigotes were washed with PBS and used immediately to infect hamster BMDMs. Cells were infected at a promastigote to macrophage ratio of 2∶1 and cultured thereafter in complete medium (CM) composed of DMEM supplemented with 1 mM sodium pyruvate (Gibco), 1× MEM amino acids solution (Sigma), 10 mM HEPES buffer (Cellgro), and 100 IU/mL penicillin/100 mg/mL streptomycin solution (Cellgro), which was supplemented with 2% heat inactivated fetal bovine serum (HIFBS). When infecting BMDMs at this ratio all parasites were internalized so that no extracellular parasites could be observed by light microscopy at 24 hrs post-infection.

### Isolation of bone marrow derived macrophages

Bone marrow cells were flushed from normal hamster femurs and adjusted to 8×10^6^/mL in RPMI with 10% HIFBS, 50 µM β-mercaptoethanol (Sigma), and supplemented with 20 ng/mL recombinant human macrophage-colony stimulating factor (M-CSF) (R&D Systems). After 3 days of culture the medium was changed and at 6–7 days of culture the cell monolayer (>95% macrophages as determined by microscopy) was washed 3 times with PBS and detached with Trypsin/EDTA (Gibco) and cell scraping. The cells were starved of M-CSF or serum in CM with 2% HIFBS overnight before the assays.

### Measurement of arginase

Arg1 expression and arginase enzymatic activity in BMDM was determined at 24 hrs or 48 hrs by real-time RT-PCR or by production of urea, respectively, as described previously [2]. For Western blot a goat anti-hamster arg1 polyclonal antibody was used [2]. The antibody used for detection of hamster arg1 did not react with *L. donovani* parasite lysates. The cells were left unstimulated or exposed to recombinant human Epidermal Growth Factor (EGF), mouse Insulin-like Growth factor-1 (IGF-1), human Platelet-Derived Growth Factor (PDGF) (Cell Signaling), human Fibroblast Growth Factor basic (heparin stabilized) (Sigma), 0.3–2.5% recombinant hamster IL-4 conditioned medium (equivalent to 3–25 IU/mL determined by STAT6 reporter bioassay) [2], or human IL-10 (R&D Systems) and/or infected with *L. donovani* promastigotes at 1∶2 macrophage∶parasite ratio. The activity of human IL-10 on hamster cells was verified using hamster BMDMs transiently transfected with a STAT3 lentiviral reporter construct (Cignal Lenti-reporter, SA Biosciences).

### Quantitative RT-PCR

Real time RT-PCR for arg1 and STAT6 mRNA was performed as described [2].

### Screening of Receptor Tyrosine Kinase (RTK) inhibitors

Spleen cells from 28-day *L. donovani* infected hamsters were cultured *ex vivo* as described previously [38] and treated for 24 hrs with each inhibitor from a library of 80 RTK inhibitors (Biomol International, Inc.) at twice the dose reported to cause 50% inhibition. Total RNA was isolated and the level of arg1 transcription determined by real time PCR as described [2].

### Screening for activated RTKs

An RTK antibody array (PathScan Array, Cell Signaling), which contains antibodies against 28 phospho-RTKs and 11 key signaling nodes of the RTK pathways, was used to identify RTKs activated by *L. donovani* infection. The mean dot-spot chemiluminescent intensity of splenic macrophages (n = 4) from infected hamsters (28 days post-infection) was compared to that of 4 uninfected hamsters by densitometry analysis (GeneTools Analysis Software, Syngene).

### Chemical inhibition of growth factor receptors

BMDMs were seeded in white clear bottom 96-well plates at 20,000 cells per well in CM and pre-treated with Fibroblast Growth Factor Receptor-1 inhibitor (PD166866; CAS 192705-79-6; Calbiochem) or Insulin-like Growth Factor Receptor inhibitor (PPP; CAS 477-47-4; Calbiochem). After 1–2 hrs the medium containing the inhibitor was discarded and the cells infected with *L. donovani* promastigotes for 20 min. Medium containing fresh inhibitors was then added back to the infected cells and the cells collected at 24 h post-infection for measurement of arg1 expression and parasite burden. Parasite load was determined by measurement of luciferase activity from luciferase-transfected parasites as described previously [38] or by real time RT-PCR using primers and a Taqman probe against the conserved sequence of the 18S gene of *Leishmania* [79] (forward primer: TTACCACCTTACGTA TCTTTTCTATTCG; reverse primer: AAAACAGAAAACGTGCTGAGG AT; Taqman probe: FAM-CT TTACCGGCCACCCACGGGA-TAMRA). Similar experiments were performed with adherent spleen cells cultured *ex vivo* from hamsters infected with *L. donovani* (21 days post-infection) as described [38]. The viability of treated cells was assessed in parallel experiments (20,000 cells/well/100 µL in 96-well white plates) by luminometric measurement of ATP (Cell Titer Glo Assay, Promega).

### Measurement of growth factor receptor activation in hamsters infected with *L. donovani*

To confirm the results of the PathScan Array, we immunoprecipitated (IP) selected growth factor receptors from fresh lysates of splenic macrophages isolated from infected hamsters using cross-reacting anti-mouse/human/rat growth factor receptor antibodies (Table S1). Following cell lysis in RIPA buffer supplemented with protease/phosphatase inhibitors (Santa Cruz) the protein concentration of total cell lysates was adjusted to 3 µg/300 µL buffer and the IP procedure was followed according the manufacturer's instructions using protein A/G agarose (Santa Cruz). In brief, pre-cleared samples were incubated with the anti-receptor antibody overnight at 4°C on an Orbital shaker, then 20 µL protein A/G agarose was added to the Ag-antibody complex and incubated for 4 hr at 4°C. The protein A/G/antibody complex was precipitated by centrifugation, washed 3 times with PBS, suspended in 50 µL of 1× LDS running buffer (Invitrogen) and the antibodies released from the agarose beads by heat (100°C, 5 min). After resolving 20 µL of sample by SDS PAGE the separated proteins were transferred to nitrocellulose membranes, blocked with TBS-T 5% milk with 1 mM sodium orthovanadate (Na_3_VO_4_) and incubated overnight at 4°C with the anti-phospho RTK in TBS-T with 0.4% BSA or TBS-T 3% milk with 1 mM Na_3_VO_4_.

### Measurement of growth factors in hamsters infected with *L. donovani*

Growth factor receptor ligands were measured in plasma or spleen homogenates from uninfected or infected hamsters. IGF-I and PDGF-β were measured by ELISA using anti-rat/mouse IGF-I and anti-rat/mouse PDGF-β using ELISA kits (R&D Systems). Epidermal Growth Factor, heparin-binding EGF-like growth factor (HB-EGF), Epiregulin and Amphiregulin were measured by immunoprecipitation/western blot using antibodies reactive against the mouse/rat/human proteins (Santa Cruz).

### Identification of activated signaling proteins

Splenic macrophages isolated by adherence from infected or uninfected hamsters were lysed and suspended in RIPA buffer containing 1× protease/phosphatase inhibitors. Lysates were stored at −80°C and used within 2 months. Ten µg of total protein was suspended in 1× LDS sample buffer and separated by SDS-PAGE in pre-cast gels (NuPage, Bis-Tris 4–12%). The separated proteins were transferred to nitrocellulose membranes using the iBlot system (20 V, 9 min) (Invitrogen). Then membranes were incubated with primary antibody (Table S1) either in TBS-T with 0.4% BSA or TBS-T with 3% milk and 1 mM Na_3_VO_4_ followed by the secondary antibody conjugated to HRP. The reaction was detected with enhanced chemiluminescent substrate (West Pico; Thermo Scientific) and captured with a Chemi X T4 camera (G BOX, SynGene) and analyzed with Gene Tools analysis Software (SynGene). The fold change of protein expression was calculated by densitometry analysis of western blot bands of infected samples (at 7, 14 and 28 days post-infection) with reference to uninfected samples.

### Transcriptional activation of STAT6

STAT6 activity was determined in the hamster BHK-21 cell line stably transfected with the luciferase reporter plasmid p(IE-IL4_RE_)_4_-LUC as described previously [2]. p-STAT6 was detected by immunoprecipitation of cell lysates (5×10^6^ cells/300 µL RIPA with phosphatase inhibitors) with 1 µg of STAT6 capture antibody (M-20, Santa Cruz Biotechnology) at 4°C. overnight. Protein A/G immunoprecipitated complexes were washed 4 times with PBS, eluted by heat 5 min 100°C in 50 µl of 1× LDS loading buffer and detected by SDS-page using anti-p-STAT6 antibody (# 9361, Cell Signaling, 1∶1000 TBS-T, 0.4% BSA, 4°C, overnight), anti-rabbit HRP conjugate, and West Pico substrate (Thermo Scientific) as above.

### Knockdown of STAT6 in hamster BMDMs

Stealth RNAi sequences were designed *in silico* using the BLOCK-iT RNAi Designer (Life Technologies) and chosen based on the sequences spanning 2 regions that were successfully targeted in knockdown of STAT6 previously [2] as follows: region 1: top, UGGCCACCAUCAGACAAAUACUUCA; bottom, UGAAGUAUUUGUCUGAUGGUGGCCA; region 2: top, CACAGUUCAACAAGGAGAUCCUGUU; bottom, AACAGGAUCUCCUUGUUGAACUGUG (each duplex synthetized and annealed by Life Technologies). Hamster BMDMs were differentiated for 6 days with 20 ng/mL of recombinant human M-CSF (R&D Systems) and plated overnight (250,000 cells per well in 24-well plates and 500 µL CM with 10% HIFBS). For transfection, 25 nM of each stealth duplex (239 and 1451) targeting hamster STAT6 was mixed in a volume of 100 µL Optimem with 0.9 µL of Lipofectamine RNAiMAX (Invitrogen) according the manufacturer's instruction. A non-targeting oligonucleotide (low GC, Invitrogen) was used as a control. Then the culture medium was discarded and 500 µL of Optimem (Invitrogen) with 10% HIFBS without antibiotics was added to the cell monolayer together with 100 µl of the transfection mix to achieve a final concentration of 8.3 nM of siRNAi oligos in 600 µL per well. The next day the transfection medium was changed for fresh Optimem with 10% HIFBS without antibiotics. At 48 hr post-transfection cells were serum starved in CM overnight, and stimulated with either *L. donovani* promastigotes or growth factors at 72 hr of transfection. Both STAT6 knockdown efficiency and arg1 transcription was measured 24 h later by real time RT-PCR and Western blot.

### Statistical analysis

Comparison between groups was typically performed using ANOVA. A parametric or non-parametric test was selected according the distribution of the raw data, followed by a post-test analysis for multiple groups (e.g. Dunnett's Multiple Comparison Test) as appropriate. Paired t test and Wilcoxon signed rank test were used to identify differences between inhibitors and vehicle controls. All analyses were conducted using GraphPad InStat version 3.00 software for Windows 95 (GraphPad Software, San Diego California USA). *P* values of <0.05 were considered significant.

## Supporting Information

Figure S1 **IGF-1 and PDGF-β production in hamsters with VL.** IGF-1 and PDGF-β proteins were measured by ELISA using anti-mouse/rat antibodies that are broadly cross-reactive across species (IGF-1 and PDGF-β generally have highly conserved sequences across species). We found no increase in their expression in serum (panels A and D), plasma (panels B and E), or spleen tissue homogenates (panels C and F) from hamsters infected with *L. donovani*. At day 7 post-infection both serum and splenic IGF-1 were significantly decreased relative to uninfected controls. By immunoprecipitation and immunoblot we were unable to detect the ligands of EGFR (EGF, HB-EGF, Epiregulin and Amphiregulin), or VEGF. These negative immunoblots are not shown. Antibodies used for these experiments were broadly reacting across multiple species, however, we cannot exclude the possibility that the lack of detection was due to an antibody that had low affinity to the hamster protein. *p<0.05; ***p<0.001.
(TIF)

Figure S2 **Expression of PDGF-β and EGFR in hamsters with VL.** Splenic macrophages were isolated by adherence from the spleens of uninfected hamsters (time 0) or hamsters infected for 7, 14, 28 or 45 days and lysates probed with antibodies directed against PDGF-β, p-PDGF-β, EGFR, p-EGFR and GAPDH (loading control). An immunoblot representative of 2–4 independent experiments is shown. Phosphorylated EGFR could not be detected with any of 3 different anti-Phospho-EGFR antibodies (Tyr1068, Tyr992, Tyr 1045; Cell Signaling); those negative immunoblots are not shown.
(TIF)

Figure S3 **Inhibitors of FGFR and IGF-1R do not affect** ***Leishmania donovani*** **viability.** Cultured *L. donovani* promastigotes were seeded in 96-well white-bottom luminometry plates at 10,000 parasites per well in DMEM with 2% heat-inactivated fetal bovine serum. The parasites were incubated at 26°C in the presence of increasing concentrations of (A) FGFR inhibitor (PD166866) or (B) IGFR inhibitor (PPP) or with vehicle control (DMSO). After 48 hours the number of viable promastigotes was determined by luminometry (cell titer Glo, Promega). Data represent the percent of viable parasites in 4 different replicates of each concentration of inhibitor compared to the control with the corresponding DMSO dilution.
(TIF)

Figure S4 **IL-10 and IL-10Rα are increased in** ***L. donovani*** **infected macrophages but are not induced by growth factors.** **A**) Expression of IL-10 and IL-10Rα mRNA was determined by qRT-PCR in splenic macrophages from uninfected (Un) and 18-day *L. donovani* infected hamsters (Inf). **B**) IL-10 and IL-10Rα mRNA in BMDMs infected 1∶2 with *L. donovani* was not amplified by exposure to IGF-1 (200 ng/mL) or FGF-2 (20 ng/mL) for 24 hrs. In fact, FGF-2 significantly decreased the expression of IL-10 and IL-10Rα mRNA in infected macrophages. Shown is mean and SEM of the fold increase of expression over uninfected, unstimulated controls from a single experiment representative of 2 independent experiments. **C**) STAT-3 reporter activity in hamster BMDM compared to human U-937 cells. Cells (10,000) were in plated in Opti-Mem 10% HIFBS and 2 µg/mL polybrene and transiently transfected with a lentiviral vector containing a STAT-3 luciferase reporter construct (20 MOI, Cignal lenti, Qiagen). 48 hrs after transfection the cells were serum starved for 24 hrs and then stimulated for 24 hrs with human IL-10 (100 ng/mL). Shown is the mean and SEM of the fold-increase of luciferase reporter activity in stimulated compared to unstimulated cells. Data are from a single experiment representative of 2 independent experiments. D) p-STAT3 detected by immunoblotting of whole cell lysates of hamster BMDM infected *in vitro* with *L. donovani* and exposed to IGF-1 (200 ng/mL) or FGF-2 (20 ng/mL) for 20 min to 48 hrs hrs. Bars represent the mean and SEM of fold change with reference to the unstimulated (Un) controls calculated by densitometry analysis of immunoblot bands from 1–3 independent experiments. *p<0.05; **p<0.01; ***p<0.001.
(TIF)

Table S1 **Antibodies used to study signaling pathways in the hamster model of visceral leishmaniasis.**
(DOCX)
