# Supplementary figures and images for: Growth Factor and Th2 Cytokine Signaling Pathways Converge at STAT6 to Promote Arginase Expression in Progressive Experimental Visceral Leishmaniasis
Source: PLoS Pathog. 2014 Jun 26;10(6):e1004165. doi: 10.1371/journal.ppat.1004165 (PMC4072777; doi:10.1371/journal.ppat.1004165)

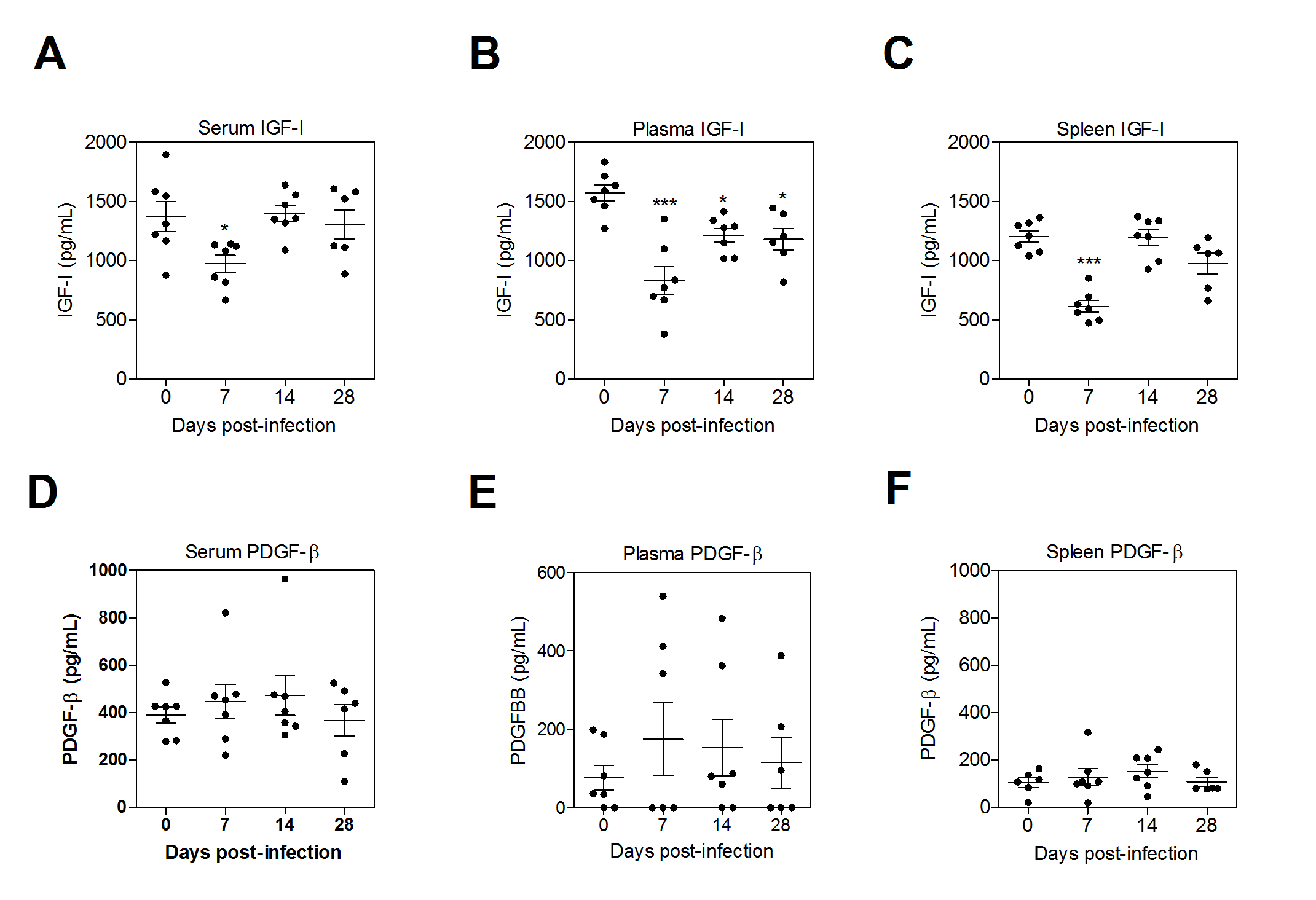

Supplement: Figure S1 — IGF-1 and PDGF-β production in hamsters with VL. IGF-1 and PDGF-β proteins were measured by ELISA using anti-mouse/rat antibodies that are broadly cross-reactive across species (IGF-1 and PDGF-β generally have highly conserved sequences across species). We found no increase in their expression in serum (panels A and D), plasma (panels B and E), or spleen tissue homogenates (panels C and F) from hamsters infected with L. donovani. At day 7 post-infection both serum and splenic IGF-1 were significantly decreased relative to uninfected controls. By immunoprecipitation and immunoblot we were unable to detect the ligands of EGFR (EGF, HB-EGF, Epiregulin and Amphiregulin), or VEGF. These negative immunoblots are not shown. Antibodies used for these experiments were broadly reacting across multiple species, however, we cannot exclude the possibility that the lack of detection was due to an antibody that had low affinity to the hamster protein. *p<0.05; ***p<0.001. (TIF) [file ppat.1004165.s001.tif]

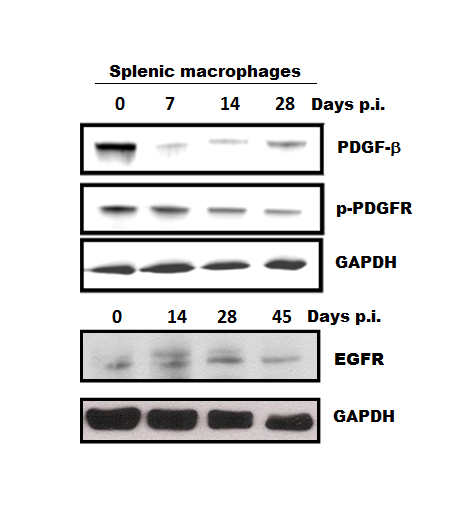

Supplement: Figure S2 — Expression of PDGF-β and EGFR in hamsters with VL. Splenic macrophages were isolated by adherence from the spleens of uninfected hamsters (time 0) or hamsters infected for 7, 14, 28 or 45 days and lysates probed with antibodies directed against PDGF-β, p-PDGF-β, EGFR, p-EGFR and GAPDH (loading control). An immunoblot representative of 2–4 independent experiments is shown. Phosphorylated EGFR could not be detected with any of 3 different anti-Phospho-EGFR antibodies (Tyr1068, Tyr992, Tyr 1045; Cell Signaling); those negative immunoblots are not shown. (TIF) [file ppat.1004165.s002.tif]

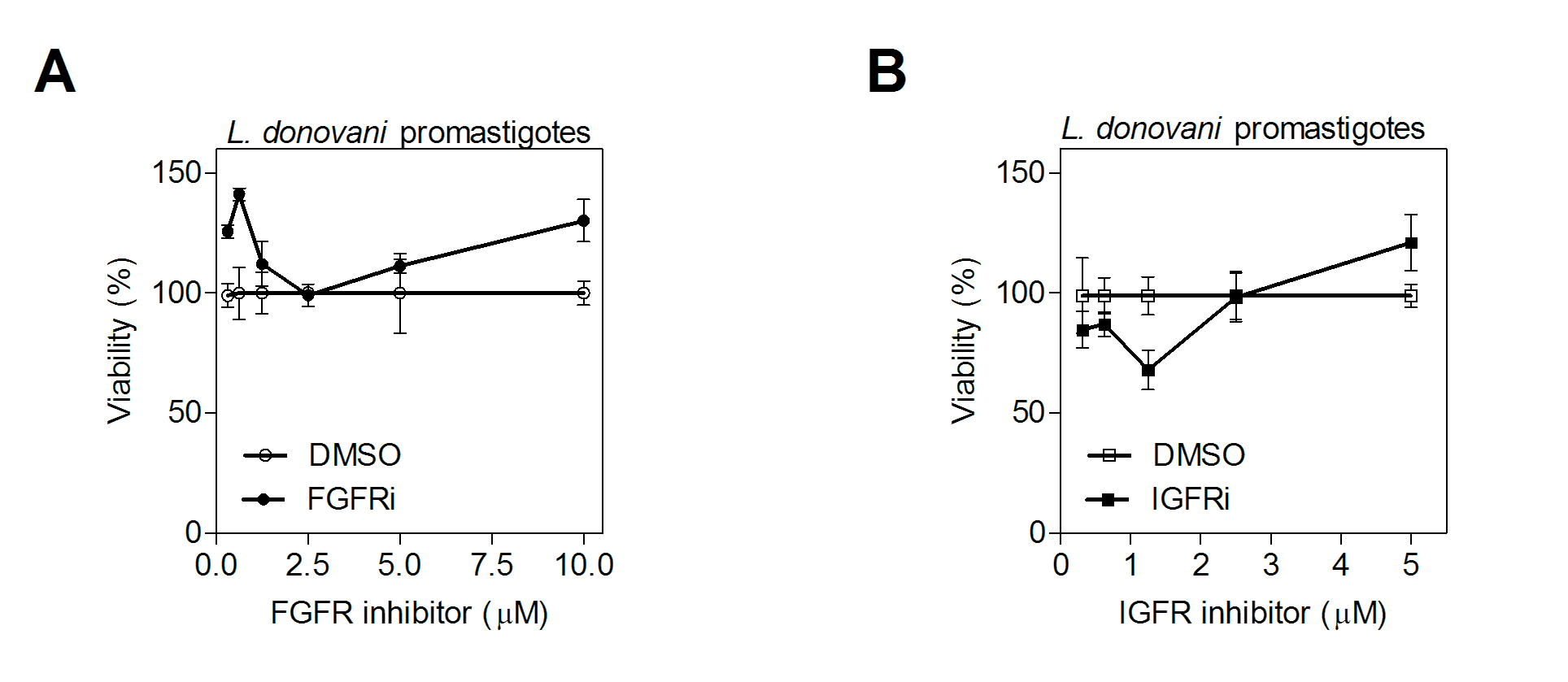

Supplement: Figure S3 — Inhibitors of FGFR and IGF-1R do not affect Leishmania donovani viability. Cultured L. donovani promastigotes were seeded in 96-well white-bottom luminometry plates at 10,000 parasites per well in DMEM with 2% heat-inactivated fetal bovine serum. The parasites were incubated at 26°C in the presence of increasing concentrations of (A) FGFR inhibitor (PD166866) or (B) IGFR inhibitor (PPP) or with vehicle control (DMSO). After 48 hours the number of viable promastigotes was determined by luminometry (cell titer Glo, Promega). Data represent the percent of viable parasites in 4 different replicates of each concentration of inhibitor compared to the control with the corresponding DMSO dilution. (TIF) [file ppat.1004165.s003.tif]

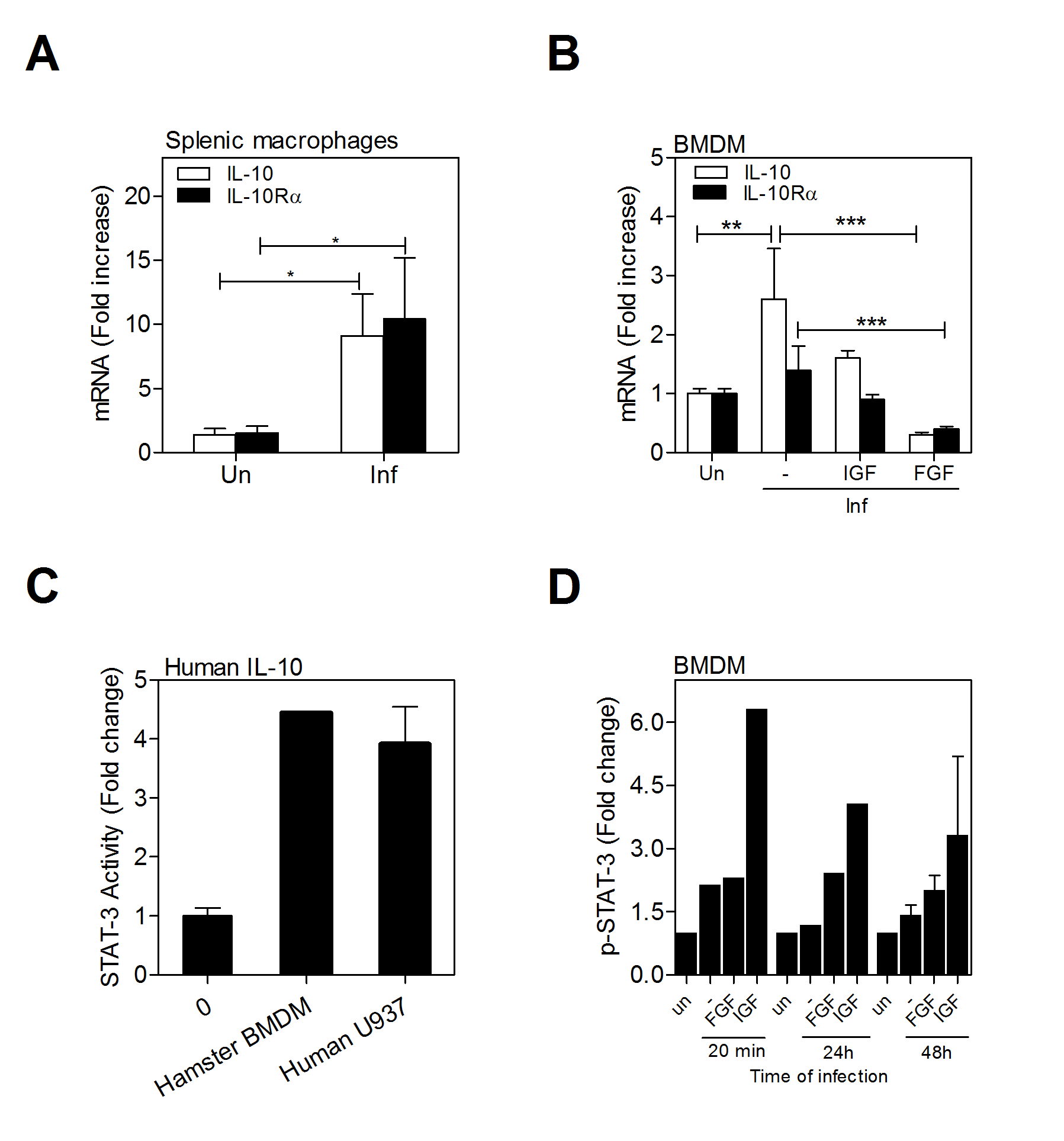

Supplement: Figure S4 — IL-10 and IL-10Rα are increased in L. donovani infected macrophages but are not induced by growth factors. A) Expression of IL-10 and IL-10Rα mRNA was determined by qRT-PCR in splenic macrophages from uninfected (Un) and 18-day L. donovani infected hamsters (Inf). B) IL-10 and IL-10Rα mRNA in BMDMs infected 1∶2 with L. donovani was not amplified by exposure to IGF-1 (200 ng/mL) or FGF-2 (20 ng/mL) for 24 hrs. In fact, FGF-2 significantly decreased the expression of IL-10 and IL-10Rα mRNA in infected macrophages. Shown is mean and SEM of the fold increase of expression over uninfected, unstimulated controls from a single experiment representative of 2 independent experiments. C) STAT-3 reporter activity in hamster BMDM compared to human U-937 cells. Cells (10,000) were in plated in Opti-Mem 10% HIFBS and 2 µg/mL polybrene and transiently transfected with a lentiviral vector containing a STAT-3 luciferase reporter construct (20 MOI, Cignal lenti, Qiagen). 48 hrs after transfection the cells were serum starved for 24 hrs and then stimulated for 24 hrs with human IL-10 (100 ng/mL). Shown is the mean and SEM of the fold-increase of luciferase reporter activity in stimulated compared to unstimulated cells. Data are from a single experiment representative of 2 independent experiments. D) p-STAT3 detected by immunoblotting of whole cell lysates of hamster BMDM infected in vitro with L. donovani and exposed to IGF-1 (200 ng/mL) or FGF-2 (20 ng/mL) for 20 min to 48 hrs hrs. Bars represent the mean and SEM of fold change with reference to the unstimulated (Un) controls calculated by densitometry analysis of immunoblot bands from 1–3 independent experiments. *p<0.05; **p<0.01; ***p<0.001. (TIF) [file ppat.1004165.s004.tif]
