# Supplementary material for: Growth Factor and Th2 Cytokine Signaling Pathways Converge at STAT6 to Promote Arginase Expression in Progressive Experimental Visceral Leishmaniasis
Source: PLoS Pathog. 2014 Jun 26;10(6):e1004165. doi: 10.1371/journal.ppat.1004165 (PMC4072777; doi:10.1371/journal.ppat.1004165)
Supplement: Table S1 — Antibodies used to study signaling pathways in the hamster model of visceral leishmaniasis. (DOCX) [file ppat.1004165.s005.docx]

**Table S1. Antibodies used to study signaling pathways in the hamster model of visceral leishmaniasis.^1^**

| **Antibody name, host** | **Symbol** | **Cat. No.** | **Supplier ^2^** |
| --- | --- | --- | --- |
| Anti-Fibroblast Growth Factor Receptor 1, Mouse, clone VBS-7 | FGFR-1 | 13-3100 | A |
| Phospho-Fibroblast Growth Factor Receptor (Tyr653/654), Mouse (55H2) | pFGFR-1 | 3474 | B |
| Anti- Epidermal growth factor receptor, Rabbit | EGFR | 06-847 | C* |
| Phospho-Epidermal Growth Factor Receptor (Tyr992), Rabbit | p-EGFR | 2235 | B |
| Platelet-Derived Growth Factor Receptor type β, Rabbit | PDGFR- β | SC-432 | D |
| Phospho-Platelet-Derived Growth Factor Receptor α (Tyr849)/β (Tyr857), Rabbit (C43E9) | p-PDGFR- β | 3170 | B |
| Insulin like-growth factor I receptor, C-20, Rabbit | IGF-IR- β | SC-713 | D |
| Phospho-Insulin like-growth factor I receptor (Tyr1161). Rabbit | p- IGF-IR- β | SC-101703 | D |
| Phospho-Signal Transducer and Activator of Transcription 6 (Tyr641), Rabbit | p-STAT-6 | 9361 | B |
| Signal Transducer and Activator of Transcription 6 | STAT-6 | SC-981 | D |
| Phospho- cAMP Responsive Element Binding Protein 1 (Ser133), Rabbit | p-CREB | 9195 | B |
| Pospho- Proto-oncogene c-Fos (Ser32) (D82C12), Rabbit | p-c-Fos | 5348 | B* |
| Arginase-1, Rabbit | Arg-1 | Custom | * |
| Phospho-Signal transducer and activator of transcription 3 (Tyr705) (D3A7), Rabbit | p-STAT-3 | 9145 | B* |
| CCAAT/enhancer-binding protein β (LAP), Rabbit | C/EBP β (LAP) | 3087 | B |
| Phospho- Insulin receptor substrate 1 (Tyr1222), Rabbit | p-IRS-1 | 3066 | B |
| Phospho- mitogen-activated protein kinase kinase 1 /2 (Ser217/221),(41G9), Rabbit | p-MEK1/2 | 9154 | B |
| Phospho- Mitogen-activated protein kinase 1/2 p44/42 MAPK (Thr202/Tyr204) (D13.14.4E), Rabbit | p-ERK1/2 | 4370 | B* |
| Phospho- Signal transducer and activator of transcription 1 (Tyr701), Rabbit | p-STAT-1 | 9171 | B |
| Phospho- Mitogen-activated protein kinase p38 (Thr180/Tyr182), Rabbit | p-p38 | 9211 | B* |
| Phospho- Proto-oncogene c-RAF (Ser338) (56A6), Rabbit | p-c-Raf | 9427 | B |
| Anti-Glyceraldehyde-3-Phosphate Dehydrogenase, clone 6C5, Mouse | GAPDH | MAB374 | C |
| Phospho- cAMP-dependent protein kinase catalytic subunit alpha (Thr197) (D45D3), Rabbit | p-PKA | 5661 | B |
| Phospho- Glycogen synthase kinase 3 beta (Ser9) (D85E12), Rabbit | p- GSK-3 β | 5558 | B* |
| Glycogen synthase kinase 3 beta (27C10), Mouse | GSK-3 β | 9832 | B* |
| Phospho- SHC-transforming protein 1 (Tyr239/240), Rabbit | p-Shc | 2434 | B |
| Anti-basic Fibroblast Growth Factor, Mouse | FGF-2 | 6110072 | E |
| Phosphatidylinositol 3-kinase p110 α (C73F8), Rabbit | PI3K p110α | 4249 | B |
| Phospho- Glycogen synthase kinase 3 alpha (Ser21), Rabbit | p- GSK-3α | 9316 | B |
| Growth factor receptor-bound protein 2, C-23, Rabbit | GRB-2 | SC-255 | D |
| Phospho-ATF-2 (Thr71) (11G2), Rabbit | p-ATF-2 | 5112 | B |
| Phospho- phosphatidylinositol 3-kinase p85 (Tyr458)/p55 (Tyr199), Rabbit | p-PIK p85 | 4228 | B |
| Ribosomal protein S6 Ser/Thr kinase p-p70 S6 kinase α (Ser 424) | p-p70 S6 | SC-293112 | D |
| Phospho-Stress-activated protein- kinase/Jun-amino-terminal kinase (G9), Mouse | p-SAPK/JNK | 9255 | B* |
| Son of sevenless 1 /2, (D-21), Rabbit | SOS ½ | SC-259 | D |
| Phospho- v-akt murine thymoma viral oncogene homolog (Ser473) (D9E), Rabbit | p-AKT | 4060 | B* |
| Phospho-c- jun oncogene (Ser63) II, Rabbit | p-c-Jun | 9261 | B |
| Protein phosphatase type 2A C Subunit (52F8), Rabbit | PP2A C | 2259 | B |
| Insulin-like Growth Factor I, Goat | IGF-I | AF791 | F |
| Insulin-like Growth Factor II, Goat | IGF-II | AF792 | F |

^1^ Most of the antibodies had reported specificity for mouse, human and rat. Cross-reaction with the hamster protein orthologs was demonstrated by reactivity to a protein of appropriate size on Western blot.

^2^ Commercial Suppliers: A: Invitrogen; B: Cell Signaling; C: Millipore; D: Santa Cruz; E: BD transduction labs; F: R&D Systems

*Reactive against hamster according the supplier.
